# Multifunctional ZnO@DOX/ICG-LMHP Nanoparticles for Synergistic Multimodal Antitumor Activity

**DOI:** 10.3390/jfb15020035

**Published:** 2024-01-30

**Authors:** Zhuoyue Li, Jingru Wang, Junwei Liu, Jianming Yu, Jingwen Wang, Hui Wang, Qingchao Wei, Man Liu, Meiqi Xu, Zhenhan Feng, Ting Zhong, Xuan Zhang

**Affiliations:** 1 Department of Pharmaceutics, School of Pharmaceutical Sciences, Peking University, Beijing 100191, China; 1410307406@pku.edu.cn (Z.L.); wjrpku@pku.edu.cn (J.W.); 2311210048@stu.pku.edu.cn (J.L.); 2010307327@stu.pku.edu.cn (J.Y.); 1810307115@pku.edu.cn (J.W.); 1810307410@pku.edu.cn (H.W.); 1910307303@pku.edu.cn (Q.W.); liuman0110@pku.edu.cn (M.L.); xmq0515@bjmu.edu.cn (M.X.); 1510307210@pku.edu.cn (Z.F.); zhongting27@bjmu.edu.cn (T.Z.); 2 Beijing Key Laboratory of Molecular Pharmaceutics and New Drug Delivery Systems, School of Pharmaceutical Sciences, Peking University, Beijing 100191, China

**Keywords:** zinc oxide, doxorubicin, indocyanine green, multifunctional nanoparticles, multimodal antitumor activity

## Abstract

Multifunctional nanoparticles are of significant importance for synergistic multimodal antitumor activity. Herein, zinc oxide (ZnO) was used as pH-sensitive nanoparticles for loading the chemotherapy agent doxorubicin (DOX) and the photosensitizer agent indocyanine green (ICG), and biocompatible low-molecular-weight heparin (LMHP) was used as the gatekeepers for synergistic photothermal therapy/photodynamic therapy/chemotherapy/immunotherapy. ZnO was decomposed into cytotoxic Zn^2+^ ions, leading to a tumor-specific release of ICG and DOX. ZnO simultaneously produced oxygen (O_2_) and reactive oxygen species (ROS) for photodynamic therapy (PDT). The released ICG under laser irradiation produced ROS for PDT and raised the tumor temperature for photothermal therapy (PTT). The released DOX directly caused tumor cell death for chemotherapy. Both DOX and ICG also induced immunogenic cell death (ICD) for immunotherapy. The in vivo and in vitro results presented a superior inhibition of tumor progression, metastasis and recurrence. Therefore, this study could provide an efficient approach for designing multifunctional nanoparticles for synergistic multimodal antitumor therapy.

## 1. Introduction

Breast cancer has high mortality rates due to its invasiveness [1,2,3,4]. Compared with chemotherapy, phototherapy has emerged as an effective approach for antitumor treatment due to its safety [5,6,7,8]. Photothermal therapy (PTT) can exert hyperthermia effects to ablate tumor cells and photodynamic therapy (PDT) can produce cytotoxic reactive oxygen species (ROS) to kill tumors [9,10]. Photosensitizers convert the surrounding triplet oxygen (^3^O_2_) into cytotoxic singlet oxygen (^1^O_2_) via direct energy transfer with the participation of oxygen (O_2_). The effect of PDT is highly dependent on the O_2_ levels at the tumor site. However, many malignant solid tumors have intrinsic characteristic hypoxia [11]. The hypoxic tumor microenvironment (TME) severely limits the PDT therapeutic efficacy [12,13]. To alleviate tumor hypoxia, there are many O_2_ supplement strategies, such as the direct delivery of exogenous oxygen and endogenous reaction to generate O_2_ in the tumor [14]. In addition, type-I PDT, which does not need O_2_, is also regarded as a choice. For example, Wang et al. designed hollow mesoporous silica nanoparticles to encapsulate O_2_-saturated perfluoropentane droplets for supplying oxygen [15]. However, O_2_ generation is greatly limited due to the loading efficiency. O_2_ self-generated nanoplatform responding to TME is more conducive for improving the antitumor efficacy of PDT. The most common method is catalyzing endogenous H_2_O_2_ to generate O_2_, since H_2_O_2_ is overexpressed in tumor tissues [16,17]. Zeng et al. designed an intelligent hollow mesoporous double shell producing O_2_ via a catalyst-like reaction to alleviate tumor hypoxia [18]. Chen et al. developed a ferroptosis inducer utilizing the Fenton reaction to provide O_2_ for PDT [19]. In our previous research, we found that zinc oxide (ZnO) could produce O_2_ to alleviate tumor hypoxia and improve efficacy [20]. ZnO NPs themselves can produce ROS as a photosensitizer for type-I PDT. The low price and good stability of the materials for ZnO have gained attention in biomedical applications, especially in drug delivery systems [21]. ZnO is pH sensitive to degrade into cytotoxic Zn^2+^ ions [22,23,24,25,26]. ZnO could be used as a pH-sensitive drug carrier.

Indocyanine green (ICG) has been approved by the FDA for PDT and PTT. Moreover, ICG also can stimulate tumor cells to release damage-associated molecular patterns to induce immunogenic cell death (ICD), which promotes dendritic cell (DC) maturation and amplifies T cell infiltration for immunotherapy [27,28,29,30,31]. The traditional first-line chemotherapy agent doxorubicin (DOX) could also trigger ICD-mediated immune responses [32,33,34,35,36]. Additionally, Yang et al. designed a DOX/ICG-loaded nanoscale ZIF-8 formation, which targeted tumors for synergistic tumor therapy [37]. Kuai et al. chose ICG and DOX loading in liposomes for cancer immunotherapy [38]. Therefore, DOX and ICG can be combined to exhibit a higher antitumor efficacy through ICG-activated PTT and PDT, DOX-derived chemotherapy and ICD-induced immunotherapy.

Herein, multifunctional ZnO@DOX/ICG-LMHP nanoparticles (ZNIDL NPs) were developed for synergistic PTT/PDT/chemotherapy/immunotherapy. The ZnO of ZNIDL NPs can be decomposed into cytotoxic Zn^2+^ ions, leading to a tumor-specific release of ICG and DOX in the acidic TME. ZnO can simultaneously produce ROS and O_2_ for PDT. The released ICG under laser irradiation could produce ROS for PDT and raise the tumor temperature for PTT. The released DOX can directly cause tumor cell death in chemotherapy. Both DOX and ICG can induce ICD in immunotherapy. Biocompatible low-molecular-weight heparin (LMHP) was used as the gatekeepers on the outside of the nanoparticles. Hence, the designed multifunctional ZNIDL NPs for synergistic multimodal antitumor activity could be an effective antitumor nano-delivery system. The ZNIDL NPs were characterized, including their morphology, drug release, and photodynamic and photothermal performance. The intracellular behavior and antitumor efficacy in vitro of the ZNIDL NPs were evaluated. The antitumor activity in vivo of the ZNIDL NPs was investigated in 4T1 tumor models. In vitro ICD signaling molecules and the in vivo DC maturation and T cell activation induced by ZNIDL NPs under laser irradiation were also investigated.

## 2. Materials and Methods

### 2.1. Materials

Indocyanine green and sodium hydroxide were obtained from Beijing Tong Guang Fine Chemicals Company (Beijing, China). Zinc acetate dehydrate was purchased from Sigma-Aldrich (St. Louis, MO, USA). Doxorubicin was obtained from Ouhe Technology Co., Ltd. (Beijing, China). Pluronic was purchased from Beijing Solable Technology Co., Ltd. (Beijing, China). The ultrafiltration tubes were purchased from Merck Millipore (Darmstadt, Germany). The RPMI 1640 cell culture medium was obtained from Macgene Biotech Co. Ltd. (Beijing, China). Fetal bovine serum was purchased from GIBCO (Carlsbad, CA, USA).

The mammary breast cancer cell line 4T1 was purchased from the Cell Resource Center, Peking Union Medial College (Beijing, China). The cells were cultured according to the recommended conditions from ATCC.

The female BALB/C mice (6 weeks) were provided by the Experimental Animal Center of the Peking University Health Science Center. The animal experimental procedures were approved by the Ethics Committee of Peking University. The project identification code was LA2021366. The date of approval was 3 June 2021.

### 2.2. Preparation of ZNIDL NPs

The sol-gel method was used to synthesize the ZnO NPs, similar to our previous report [20]. The compound 3-aminopropyltrimethoxysilane was used to modify the surfaces of the ZnO NPs to form ZnO-NH_2_ NPs with a more hydrophilic surface [39]. The ZnO-NH_2_ NPs, DOX and ICG-LMHP solution were incubated overnight to form the mixture, which was re-dissolved in dimethyl sulfoxide and added dropwise into distilled water with rapid stirring. Finally, the ZnO@DOX/ICG-LMHP nanoparticles (ZNIDL NPs) were obtained from zinc oxide nanoparticles loading doxorubicin and indocyanine green modified by low-molecular-weight heparin.

### 2.3. Characterization of ZNIDL NPs

The ZNIDL NPs were characterized, including the particle size, morphology and spectrum, using a dynamic light scattering detector (Malvern, Worcestershire, UK), transmission electron microscope (JEOL, Tokyo, Japan), Fourier transform infrared spectrometer (ThermoFisher, Waltham, MA, USA), X-ray powder diffractometer (Panalytical, Alemlo, The Netherlands), UV–Vis spectrophotometer (Mapada, Shanghai, China) and fluorescence spectrophotometer (Shimadzu, Kyoto, Japan).

### 2.4. Drug Release of ZNIDL NPs

The pH-sensitive and photo-stimulated behavior of the ZNIDL NPs were investigated in solution by directly adding a buffer of different pHs under laser irradiation for different times. The ZNIDL NPs were also added into the dialysis bag and immersed into a medium with different pHs under laser irradiation at selected 2, 4 and 6 h time points [40,41,42]. The fluorescence intensity was determined, and the released rates of DOX were calculated.

### 2.5. Oxygen Generation of ZNIDL NPs

The oxygen generation in the mixed ZnO NPs and H_2_O_2_ solution was recorded using the dissolved oxygen meter. Meanwhile, the fluorescence of the O_2_ sensing probe (Ru(dpp)_3_)Cl_2_, which was reduced by molecular oxygen, was recorded using in vivo imaging systems. All the solutions were pre-saturated with nitrogen to remove dissolved oxygen.

### 2.6. Photodynamic and Photothermal Effect of ZNIDL NPs

Dichloro-dihydro-fluorescein (DCFH) was used as a probe to detect the total ROS generation [43,44,45]. All the solutions were irradiated for various times. Singlet oxygen (^1^O_2_) was detected using 1,3-diphenylisobenzofuran (DPBF). The fluorescence intensity was determined using fluorescence spectrophotometry. The temperature changes of the different ICG concentrations for the given laser irradiation and the different power for the given concentrations and different samples were recorded using a thermal infrared imaging camera (Fotoric, Shanghai, China).

### 2.7. Intracellular Behavior of ZNIDL NPs

The ZNIDL NPs and DOX incubated with 4T1 cells at a concentration of 8 μM DOX. The DOX fluorescence intensity was evaluated using a confocal laser scanning microscope (ZEISS, Oberkochen, Germany) and a flow cytometer (Beckman Coulter, Brea, CA, USA). The probe dichloro-dihydro-fluorescein diacetate (DCFH-DA) was used to estimate the ROS within the 4T1 cells. The fluorescence intensity was detected using a confocal laser scanning microscope and a flow cytometer. 

### 2.8. In Vitro Antitumor Activity of ZNIDL NPs

The Sulforhodamine B (SRB) assay was used to evaluate the antitumor activity of the ZNIDL NPs in vitro, as previously reported [46,47]. The absorbance was detected using the ELx808 plate reader (BioTek, Winooski, VT, USA). The Annexin V-FITC/DAPI Apoptosis Kit was used to evaluate cell apoptosis via flow cytometer. The cell viability and apoptosis rate were calculated.

### 2.9. ICD Signaling Molecules of ZNIDL NPs

Immunogenic cell death (ICD) could stimulate tumor cells to release damage-associated molecular patterns, including the cell surface exposure of calreticulin (CRT), adenosine triphosphate (ATP) and the extracellular release of high-mobility group box-1 (HMGB1). According to the manufacturer instructions, the CRT antibody, a Kinase-Lumi TM Lumi-nescent Kinase Assay Kit and an HMGB1 ELISA Kit were used to investigate the release of CRT, ATP and HMGB1, respectively.

### 2.10. In Vivo Antitumor Activity of ZNIDL NPs

The female BALB/c mice were subcutaneously inoculated with a 1 × 10^6^ 4T1 cells suspension. When the tumor volume reached approximately 100 mm^3^, the mice were randomly assigned to four groups and treated with (1) Blank gel, (2) ZNIDL gel, (3) ICG gel + Laser, (4) ZNIDL gel + Laser at an ICG dose of 1.5 mg/kg. The gel was prepared with pluronic, similar to our previous report via peri-tumoral injection [48]. The time was 5 min, and the power was 1.0 W/cm^2^ for laser irradiation. Calipers were used to measure the tumor diameter, and the tumor volumes were calculated using the following formula. V = length (cm) × width^2^ (cm^2^) × 0.5236. At the end of the experiment, all the mice were euthanized, and the harvested tumors were subjected to terminal deoxynucleotidyl transferase dUTP nick end labeling (TUNEL) and the hypoxia-inducible factor (HIF)-1α antibody for histopathological analysis. The main organs were collected using hematoxylin and eosin (H&E) to detect the histological changes. Throughout the study, the survival time and weight were monitored.

### 2.11. In Vivo Antitumor Immune Response of ZNIDL NPs

The animal model was consistent with the above antitumor activity in vivo. The mice were sacrificed at a specified time. The tumor, spleens and lymph nodes were collected for further testing. The release of CRT and HMGB1 induced by the ZNIDL gel in vivo were investigated. The anti-mouse MHCII-PE, CD11c-FITC and CD86-APC antibodies were used to detect activated DCs in the tumor-draining lymph nodes. The anti-mouse CD3e-APC, CD4-FITC, CD8a-APC-eFluor780 and CD25-PE antibodies were used to detect activated T cells in the tumors and spleens.

### 2.12. In Vivo Antitumor Abscopal Effect of ZNIDL NPs

The bilateral 4T1 tumor model was used to evaluate the abscopal antitumor effect of ZNIDL NPs under irradiation. In brief, primary tumors were inoculated on the right side (1 × 10^6^ 4T1 cells per mouse) and distant tumors were inoculated in the left side (2 × 10^5^ 4T1 cells per mouse) after 6 days. Finally, all the primary and distant tumor weights were measured [29].

### 2.13. Postoperative Tumor Recurrence Model of ZNIDL NPs

Tumor resection was conducted as follows [49,50,51]. The female BALB/c mice were subcutaneously inoculated with a 4T1-luc cell suspension (1 × 10^6^ cells). When the tumors were approximately 200 mm^3^ in size, primary tumors were partially removed, leaving 10% residual tissue behind. The tumor development was evaluated using in vivo imaging systems.

### 2.14. Statistical Analysis

The data were expressed as the mean ± standard deviation (SD). *T*-tests were used to determine the statistical significance. All the statistical analyses were performed using GraphPad Prism (GraphPad software version 8, La Jolla, CA, USA).

## 3. Results and Discussion

### 3.1. Characterization of ZNIDL NPs

**Figure 1:**
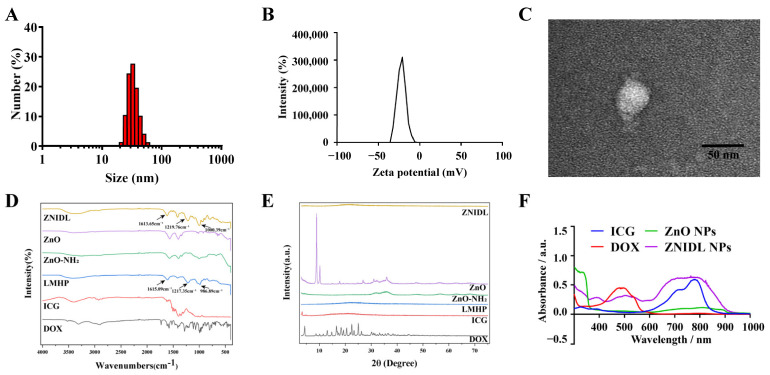
Characterization of the ZNIDL NPs. (**A**). ZNIDL NP size. (**B**). ZNIDL NP zeta potentials. (**C**). ZNIDL NP morphology. Scale bars = 50 nm. (**D**). ZNIDL NP Fourier transform infrared spectroscopy spectra. (**E**). ZNIDL NP powder X-ray diffraction spectra. (**F**). ZNIDL NP UV–Vis absorption spectra.

ZnO, DOX and the ICG and LMHP solution could self-assemble to form ZnO@DOX/ICG-LMHP nanoparticles (ZNIDL NPs). ZNIDL NPs were formed through electrostatic interactions between the amino groups in DOX, the sulfonic groups in ICG, the amino group in ZnO and the carboxyl groups in LMHP. As shown in Figure 1A,B, the particle size of the ZNIDL NPs was 34.04 ± 0.31 nm and PDI was 0.17 ± 0.013. The zeta potential of the ZNIDL NPs was −24.87 ± 0.06 mV. The morphology of the ZnO-NH_2_ NPs was consistent with the ZnO NPs showing a hexagonal crystal form, as presented in Supplementary Material Figures S1 and S2. The interior of the ZNIDL NPs consisted of some small ZnO NPs, forming a core–shell structure, as shown in Figure 1C and Figure S3. As shown in Figure 1D, the infrared characteristic peaks of the carboxyl, sulfonic and sulfonamide groups of the ZNIDL nanoparticles located at 1613.65 cm^−1^, 1219.76 cm^−1^ and 1000.39 cm^−1^ were basically consistent with those of LMHP, showing that LMHP existed on the surface of the ZNIDL NPs. The ZNIDL NPs profile was basically consistent with that of LMHP, and no crystal peaks of ZnO and DOX were observed, as shown in Figure 1D, indicating that LMHP was on the surface of the ZNIDL NPs. There were three obvious absorption peaks from the ZnO NPs at 400 nm, DOX at 500 nm and ICG at 780 nm in the ZNIDL NPs (Figure 1F). In the fluorescence spectrum, the characteristic emission peak of the ZNIDL NPs showed similar results. The emission peak intensity of ICG at 808 nm was independent of the amount of zinc ions added but was directly proportional to the amount of ZnO NPs and ZnO-NH_2_ NPs added, which indicated that ICG and the ZnO NPs interacted with each other and could be combined (Figures S4 and S5).

**Figure 2:**
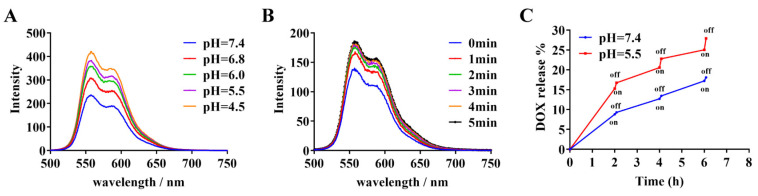
Characterization of the ZNIDL NPs. (**A**). ZNIDL NP fluorescence spectra at different pHs. (**B**). ZNIDL NP fluorescence emission spectra with laser irradiation. (**C**). In vitro DOX release from the ZNIDL NPs.

The fluorescence intensity gradually increased as the pH decreased, indicating that the ZNIDL NPs were pH sensitive, as shown in Figure 2A. Irradiation promoted the release of DOX from the ZNIDL NPs (Figure 2B). The reason was that the increased temperature caused by laser irradiation could destroy the electrostatic interaction between the sulfonate groups in ICG and the amine groups in DOX [52]. This was also demonstrated by the determination of the DOX release using dialysis methods. The cumulative DOX release from the ZNIDL NPs at pH 7.4 and pH 5.5 was 20.84% and 32.72%, respectively, at 37 °C at the final time point, as shown in Figure S6. As the temperature increased, the cumulative DOX release gradually increased. Laser irradiation significantly increased the DOX release. (Figure 2C). In the acidic tumor microenvironment and photothermal treatment, the ZNIDL NPs released more chemotherapeutic DOX, which reduced the toxicity of the ZNIDL NPs to normal tissue.

We previously confirmed that ZnO NPs could produce O_2_ through the Haber–Weiss reaction, disproportional reaction and Fenton reaction to alleviate tumor hypoxia and normalize the tumor vessels [20]. ZnO could catalyze H_2_O_2_ to produce some reactive intermediates, including a hydroxyl radical (^•^OH) and a superoxide anion (O_2_^•−^) (2H_2_O_2_ → O_2_^•−^ + 2^•^OH + 2H^+^). O_2_^•−^ could be catalyzed, such as in the Haber–Weiss reaction, to react with H_2_O_2_ (O_2_^•−^ + H_2_O_2_ → O_2_ + ^•^OH +HO^−^) or in the disproportional reaction to generate H_2_O_2_ (O_2_^•−^ + H^+^ → H_2_O_2_ + O_2_). It could also participate in the Fenton reaction with Fe^3+^ (Fe^3+^ + O_2_^•−^ → Fe^2+^ + O_2_) to generate O_2_ [14]. As shown in Figure S7, obvious bubbles were observed in the ZnO within the H_2_O_2_ group while negligible bubbles were found in the H_2_O_2_ group, indicating that ZnO NPs could produce oxygen (O_2_). In addition, we verified the results using an O_2_ sensing probe (Ru(dpp)_3_)Cl_2_ (Figure S8) [53,54,55]. Therefore, we used the ZnO NPs as a carrier to load ICG, providing O_2_ for the photodynamic process to relieve tumor hypoxia and improve antitumor activity.

**Figure 3:**
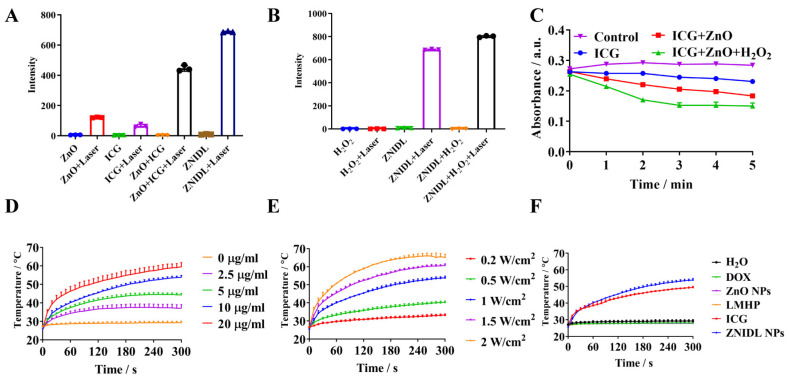
Photodynamic and photothermal performance of ZNIDL NPs. (**A**). ROS generation of the ZNIDL NPs under laser irradiation using dichloro-dihydro-fluorescein (DCFH) as an indicator. (**B**). ROS generation of the ZNIDL NPs with H_2_O_2_. (**C**). Singlet oxygen generation by DPBF. (**D**). Temperature increase profiles for the ZNIDL NPs at different concentrations with the power of 1 W/cm^2^. (**E**). Temperature increase profiles for the ZNIDL NPs with different power intensities at the ICG concentration of 10 μg/mL. (**F**). Temperature increase profiles for the ZNIDL NPs with the power of 1 W/cm^2^ at the ICG concentration of 10 μg/mL with H_2_O, DOX, ZnO NPs, LMHP and ICG used as the control samples.

It was reported that, according to the photophysical and photochemical bases, PDT included type-I and type-II [14]. Type-II PDT relied on O_2_ at the tumor site, while type-I PDT could drive therapy normally in hypoxic tumors [56]. Zhang et al. reported a core–shell structure, Ce^III^-doped LiYF_4_@SiO_2_@ZnO, enabling the generation of electron–hole pairs in the ZnO NPs to form biotoxic hydroxyl radicals for type I PDT [57]. Metal oxide nanomaterials, such as ZnO NPs, hold great potential as photosensitizers due to their excellent photostability and ability to generate radicals [58]. As shown in Figure 3A,B, ZnO NPs themselves produced ROS as a photosensitizer for type-I PDT. The ZNIDL NPs with laser irradiation induced ROS upregulation, which was significantly higher than ICG alone. ZnO provided O_2_ for the ICG O_2_-consuming processes and enhanced the total amount of ROS produced. Meanwhile, the heterostructure of ICG combined with ZnO retarded the recombination of the photogenerated electron–hole pairs to exhibit a potent PDT performance [55]. Finally, singlet oxygen (^1^O_2_) was evaluated using DPBF. The fluorescence of DPBF decreased upon treatment with ICG and ZnO with H_2_O_2_ under laser irradiation (Figure 3C), indicating that ZNIDL NPs have an excellent potential to produce ROS.

As expected, the ZNIDL NPs displayed strong photothermal effects since ICG had strong near-infrared absorption (Figure 3D–F). Temperature increases dependent on the concentration and laser power intensity were observed. The temperature of the ZNIDL NPs increased by approximately 28 °C irradiation, consistent with the ICG suspension, while the control samples, including H_2_O, DOX, ZnO and the LMHP solution, only had a temperature change of less than 2 °C. The photostability of the ZNIDL NPs was measured over five cycles of irradiation (Figures S9 and S12). According to these results, ZNIDL NPs may be employed as an efficient phototherapy agent.

### 3.2. Intracellular Behavior of ZNIDL NPs

**Figure 4:**
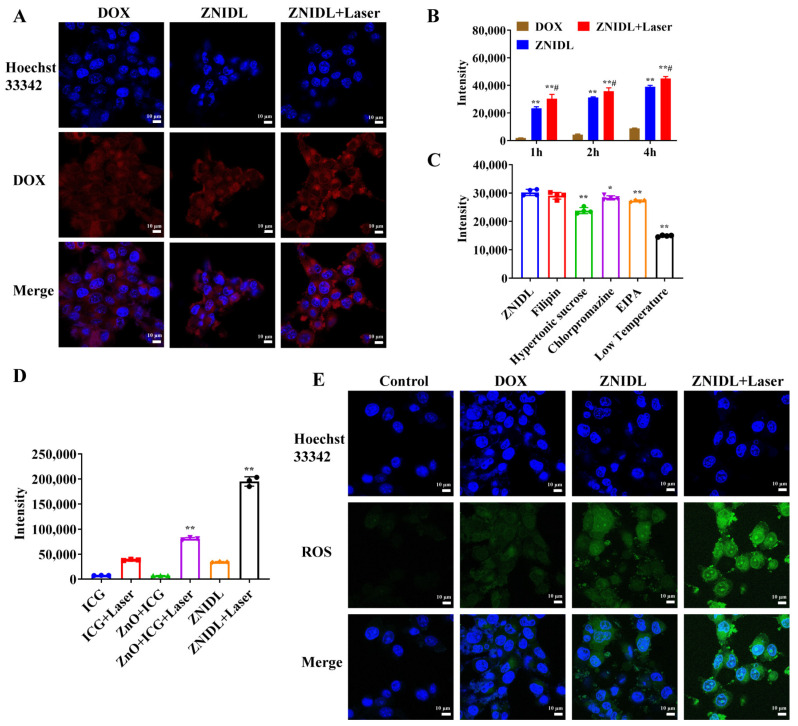
Intracellular behavior of ZNIDL NPs. (**A**). DOX cellular uptake of ZNIDL NPs in 4T1 cells. (**B**). Quantitative analysis of the DOX cellular uptake of ZNIDL NPs in 4T1 cells. ** *p* < 0.01 vs. the DOX treatment group. ## *p* < 0.01 vs. the ZNIDL treatment group. (**C**). Endocytosis pathways of ZNIDL NPs in 4T1 cells. * *p* < 0.05 vs. the control treatment group. ** *p* < 0.01 vs. the control treatment group. (**D**). Intracellular ROS generation in 4T1 cells ** *p* < 0.01 vs. the ICG treatment group. (**E**). ROS distribution in 4T1 cells.

ZNIDL + Laser had an obvious stronger DOX fluorescence than the ZNIDL NPs and free DOX, indicating that the ZNIDL NPs had a higher cellular uptake (Figure 4A). The photothermal effects induced by the ZNIDL NPs under laser irradiation enhanced the cellular uptake by increasing the fluidity of the cell membrane [59,60]. Flow cytometry had similar results (Figure 4B). The uptake of the ZNIDL NPs was time dependent. The uptake of the ZNIDL NPs decreased in the hypertonic sucrose, chlorpromazine and EIPA groups while there was no difference in the filipin group, as shown in Figure 4C. The uptake of the ZNIDL NPs was through clathrin-mediated endocytosis and macropinocytosis. The decreased uptake at low temperatures indicated that the cellular uptake was energy dependent. As stated above, the results of ROS in solution, intracellular ROS produced by ZnO + ICG + Laser and ZNIDL + Laser were nearly 2.1 and 5.1 times higher than ICG + Laser, respectively, indicating that the ZNIDL NPs generated a high level of ROS due to the existence of ZnO (Figure 4D,E and Figure S11).

**Figure 5:**
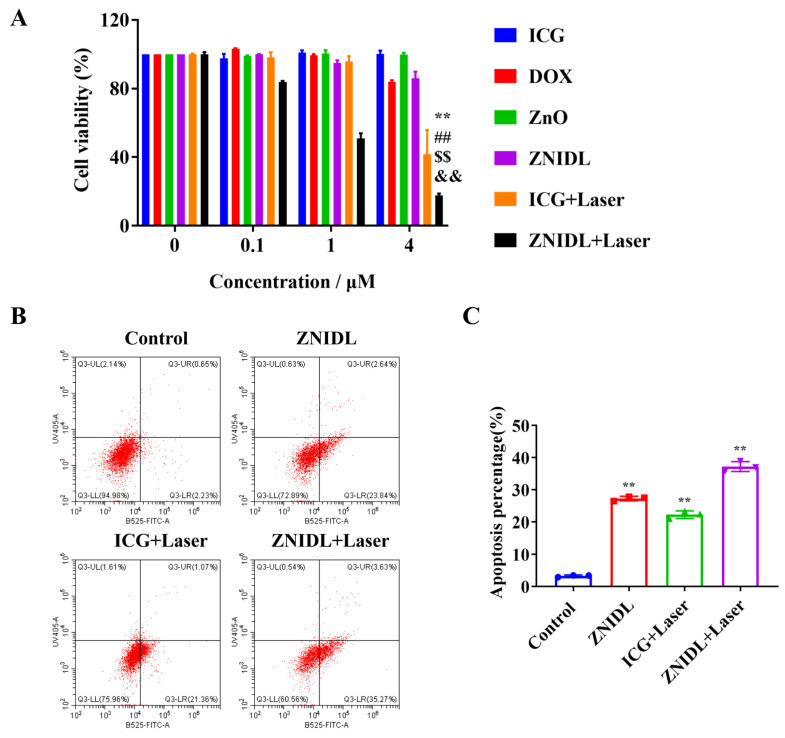
In vitro antitumor activity of ZNIDL NPs. (**A**). In vitro cell viability treated with ZNIDL NPs in the 4T1 cells. ** *p* < 0.01 vs. the ICG treatment group. ## *p* < 0.01 vs. the DOX treatment group. $$ *p* < 0.01 vs. the ZnO treatment group. && *p* < 0.01 vs. the ZNIDL treatment group. (**B**). Apoptosis in the 4T1 cells induced by ZNIDL NPs. (**C**). Quantitative analysis of apoptosis in the 4T1 cells induced by ZNIDL NPs. ** *p* < 0.01 vs. the control treatment group.

The antitumor effect was gradually enhanced with the dosage increase from 0.1 to 4 µg/mL using the SRB assay in all cases, as shown in Figure 5A. ICG had a main contribution in both PDT and PTT. The degradation of ZnO into cytotoxic Zn^2+^ ions and the temperature increase in ICG under laser irradiation promoted the release of DOX in the ZNIDL NPs and improved the antitumor efficacy. The apoptosis rates in the 4T1 cells are shown in Figure 5B,C. The total cell apoptosis rates in the ZNIDL + Laser treatment groups were 38.90% and the apoptosis level was the highest, which came from ICG + Laser and the ZNIDL NPs.

### 3.3. In Vitro Evaluation of the Immunogenic Cell Death Effect

**Figure 6:**
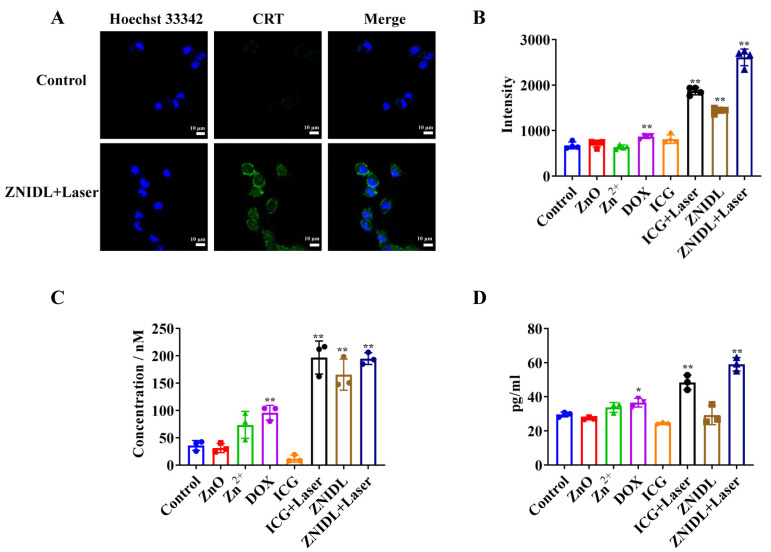
ICD signaling molecules of ZNIDL NPs. (**A**). CRT levels on the surfaces of the 4T1 cells with ZNIDL NPs using confocal laser scanning microscopy. (**B**). CRT levels of the 4T1 cells with ZNIDL NPs using flow cytometry. (**C**). Released ATP from the 4T1 cells with ZNIDL NPs. (**D**). Released HMGB1 from the 4T1 cells with ZNIDL NPs. * *p* < 0.05 vs. the control treatment group. ** *p* < 0.01 vs. the control treatment group.

Immunogenic cell death (ICD) stimulated tumor cells to release damage-associated molecular patterns (DAMPs), including the cell surface exposure of calreticulin (CRT), extracellular release of adenosine triphosphate (ATP) and high-mobility group box-1 (HMGB1) for antitumor immune response [29]. Some chemotherapy agents such as DOX and photosensitizers such as ICG induced ICD effects [61,62,63]. As shown in Figure 6A,B, the cells treated with ZNIDL + Laser exhibited higher CRT exposure levels than the other groups. In addition, HMGB1 and ATP induced by the ZNIDL NPs under laser irradiation reached the highest expression levels, showing the strongest ICD effect caused by PDT, PTT and chemotherapy (Figure 6C,D).

### 3.4. In Vivo Antitumor Activity of ZNIDL NPs

**Figure 7:**
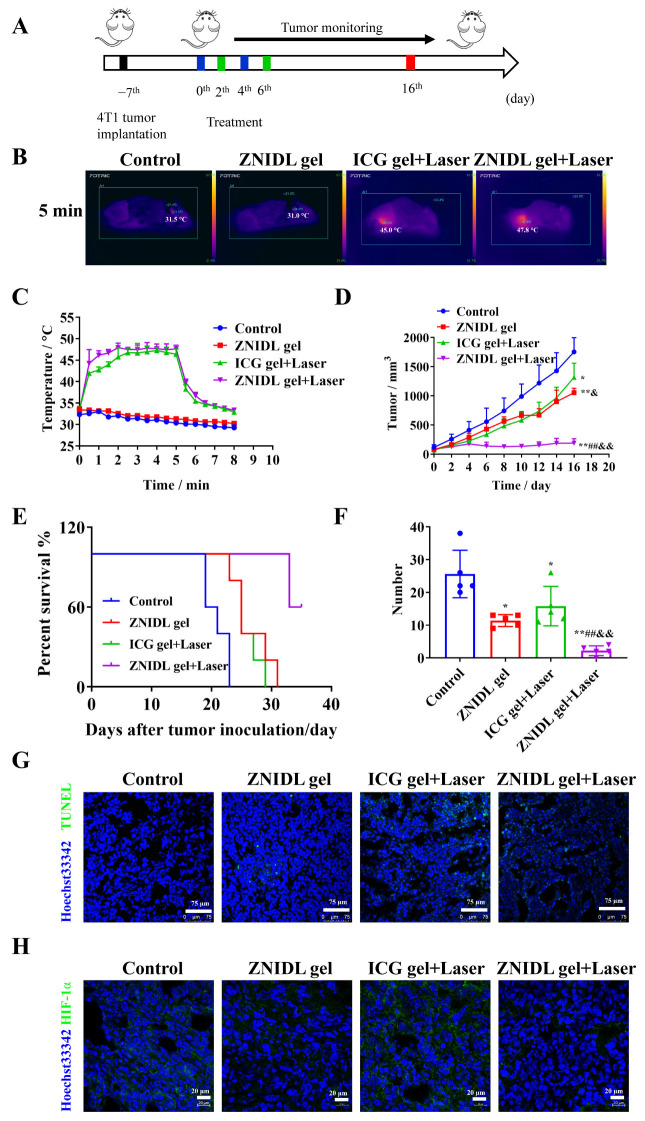
In vivo antitumor activity of ZNIDL NPs. (**A**). Scheme for the 4T1 tumor-bearing BALB/c mice model. (**B**). In vivo photothermal images under laser irradiation. (**C**). Temperature changes of the tumor in mice via peri-tumoral injection with the ZNIDL NPs under laser irradiation. (**D**). The tumor growth curves (n = 5). (**E**). The survival curves (n = 5). (**F**). Quantification of the pulmonary nodules (n = 5). (**G**). TUNEL staining. Scale bars = 100 μm. (**H**). HIF-1α immunofluorescence staining. Scale bars = 20 μm. * *p* < 0.05 vs. the control treatment group. ** *p* < 0.01 vs. the control treatment group. ## *p* < 0.01 vs. the ZNIDL gel treatment group. & *p* < 0.05 vs. the ICG gel + Laser treatment group. && *p* < 0.01 vs. the ICG gel + Laser treatment group.

The 4T1 tumor-bearing BALB/c mice model was established to investigate the antitumor activity of ZNIDL NPs in vivo (Figure 7A). Local therapy, such as peri-tumoral administration with high safety, not only led to local tumor destruction but also inhibited distant metastasis via ICD effects [30,64,65,66,67]. Injectable hydrogels had a uniform porous network structure, which was conducive to the encapsulation and controlled release of small molecule drugs [68]. The gel was prepared with pluronic, similar to our previous report [48]. The gel was temperature sensitive and formed quickly at 37 °C. As shown in Figure S12, it had a good gel dissolution ability. The temperature of the tumor in the mice treated with the ZNIDL gel increased to 47.8 °C for first laser irradiation and to 45.1 °C for the second laser irradiation. Then, it returned to the original temperature within 3 min while the control group displayed the normal physical temperature at the tumor site (Figure 7B,C and Figures S13 and S14), confirming that ZNIDL gel could be used for PTT.

The tumor volumes at the end are presented in Figure 7D. The mean tumor sizes in the ZNIDL gel + Laser, ICG gel + Laser, ZNIDL gel treatment groups and control group were 189.5 ± 75.35, 1318 ± 243.2, 1054 ± 77.30 mm^3^ and 1755 ± 242.8 mm^3^, respectively. The tumor growth inhibition rates in the ZNIDL gel + Laser, ICG gel + Laser and ZNIDL gel treatment groups were 89.20%, 24.90% and 39.94% compared to the control group. The ZNIDL gel + Laser treatment group significantly inhibited 4T1 tumor growth owing to DOX for chemotherapy and ICG for PDT and PTT. The individual tumor growth curves and survival curves presented significant differences on tumor suppression (Figure S15 and Figure 7E). Lung tissues were collected, and more pulmonary nodules were observed in the other treatment groups than the ZNIDL gel + Laser treatment group, as shown in Figure S16 and Figure 7F. These results indicated that ZNIDL NPs not only suppressed tumor proliferation but also reduced tumor metastasis in vivo.

The ZNIDL gel + Laser treatment group exhibited more potent apoptosis inhibition than the other treatment groups in TUNEL staining (Figure 7G and Figure S17). In addition, the less hypoxic areas in HIF-1α staining were observed in the ZNIDL gel and ZNIDL gel + Laser treatment group (Figure 7H and Figure S18), confirming that ZnO could markedly alleviate tumor hypoxia. There were no obvious histopathological changes in hematoxylin-eosin staining observed in the main organs, while the lungs of the control group showed some nodules (Figure S19). Additionally, all the treatment groups had no obvious weight loss, as shown in Figure S20.

### 3.5. In Vivo Antitumor Immune Response of ZNIDL NPs

**Figure 8:**
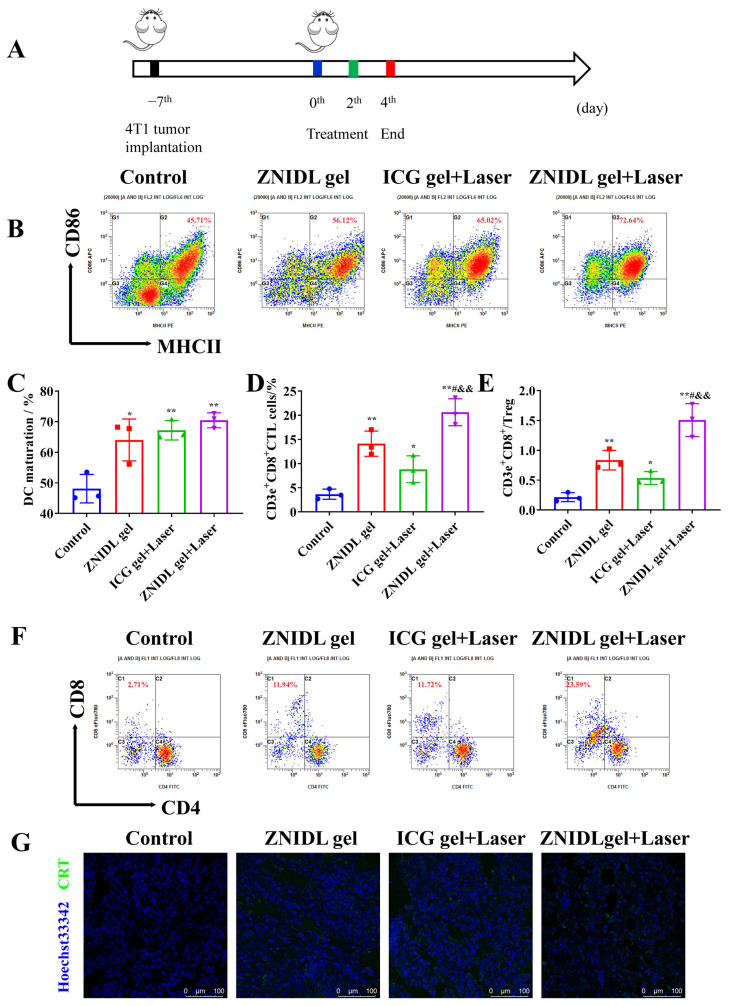
In vivo antitumor immune response of ZNIDL NPs. (**A**). Scheme of the 4T1 tumor-bearing BALB/c mice model. (**B**). Representative scatter plots of DCs in inguinal lymph nodes using flow cytometry. (**C**). Percentages of mature DCs in inguinal lymph nodes. (**D**). Percentages of CD3^+^ CD8^+^ T cells in tumors. (**E**). Ratio of CD8^+^ T cells to Tregs in tumors. (**F**). Representative scatter plots of CD8^+^ T cells in tumors using flow cytometry. (**G**). CRT immunofluorescence imaging in tumors. Scale bar = 100 μm. * *p* < 0.05 vs. the control treatment group. ** *p* < 0.01 vs. the control treatment group. # *p* < 0.05 vs. the ZNIDL gel treatment group. && *p* < 0.01 vs. the ICG gel + Laser treatment group.

Immunotherapy, which promotes dendritic cell (DC) maturation and amplifies T cells infiltration in vivo through ICD signaling molecules, was evaluated, as shown in Figure 8A. We collected and detected immune cells in the lymph nodes and tumor tissues. The gating strategy of the DCs and T cells expression levels is shown in Figures S21 and S22. As shown in Figure 8B,C, ZNIDL gel + Laser had more mature DCs in the tumor-draining lymph nodes than the control group. In addition, the ZNIDL gel + Laser treatment group triggered the highest percentages of CD8^+^ T cell and CD8^+^/Treg ratio infiltration among all the groups (Figure 8D–F and Figure S23). The results showed that the treatment using the ZNIDL gel under laser irradiation robustly promoted the infiltration of DCs in the lymph nodes and CD8^+^ T cells in the tumors and spleens, indicating that the ZNIDL gel could efficiently activate antitumor immunity. Meanwhile, immunofluorescence staining displayed the most significant CD8^+^ T cell recruitments in the ZNIDL gel +Laser treatment group, consistent with the results from flow cytometry (Figure S24). The ZNIDL gel + Laser treatment group showed a higher CRT exposure and HMGB1 release in the tumor section immunofluorescence images (Figure 8G and Figures S25 and S26). All the results indicated that ZNIDL NPs could prime ICD effects in the primary tumor and induce favorable antitumor immunity, including DC maturation and T cell activation for distant tumor inhibition.

### 3.6. Abscopal Effects and Antitumor Recurrence of ZNIDL NPs

**Figure 9:**
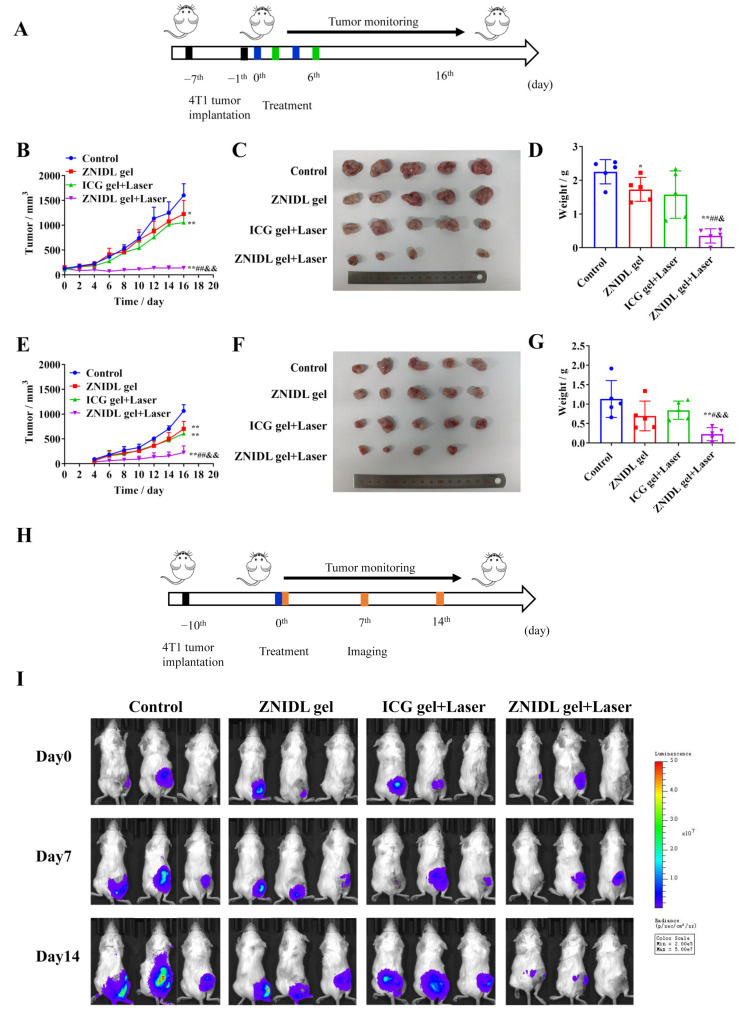
Abscopal effects and antitumor recurrence of ZNIDL NPs in vivo. (**A**). Scheme of the 4T1 bilateral tumor model. (**B**). Average primary tumor growth curves (n = 5). (**C**). Photographs of primary tumors. (**D**). Tumor weight of primary tumors. (**E**). Average distant tumor growth curves (n = 5). (**F**). Photographs of distant tumors. (**G**). Tumor weight of distant tumors. (**H**). Scheme of the 4T1 postoperative tumor recurrence model. (**I**). Images of postoperative mice in vivo imaging systems. * *p* < 0.05 vs. the control treatment group. ** *p* < 0.01 vs. the control treatment group. # *p* < 0.05 vs. the ZNIDL gel treatment group. ## *p* < 0.01 vs. the ZNIDL gel treatment group. & *p* < 0.05 vs. the ICG gel + Laser treatment group. && *p* < 0.01 vs. the ICG gel + Laser treatment group.

The bilateral 4T1 tumor model was established to observe the abscopal effects of ZNIDL NPs (Figure 9A). The tumor volumes are presented in Figure 9B,E. The mean primary tumor sizes at the end in the ZNIDL gel + Laser, ICG gel + Laser, ZNIDL gel treatment groups and control group were 140.5 ± 35.72, 1054 ± 183.4 and 1226 ± 122.7 mm^3^ and 1602 ± 232.9 mm^3^, respectively. The tumor growth inhibition rates in the ZNIDL gel + Laser, ICG gel + Laser and ZNIDL gel treatment groups were 91.23%, 34.21% and 23.47% compared to the control group. The mean distant tumor sizes at the end in the ZNIDL gel + Laser, ICG gel + Laser, ZNIDL gel treatment groups and control group were 223.7 ± 135.8, 607.5 ± 76.86, 702.8 ± 151.3 mm^3^ and 1064 ± 126.2 mm^3^, respectively. The corresponding tumor growth inhibition rates in the ZNIDL gel + Laser, ICG gel + Laser and ZNIDL gel treatment groups were 78.98%, 42.90% and 33.95% compared to the control group. The individual curve also presented significant differences on tumor suppression (Figure S27). The tumor tissue images and tumor weights also indicated an enhanced antitumor efficacy for the ZNIDL gel under laser irradiation (Figure 9C,D,F,G). There was no obvious weight loss in the mice in all the groups during the observation time, as shown in Figure S28. Taken together, ZNIDL NPs can not only kill primary tumors but also suppress the growth of distant tumors promoting a strong immune response.

Surgical resection is the first-line strategy in the current clinical treatment for breast cancer. However, surgery is incomplete as residual microtumors could lead to tumor recurrence [69,70]. Wang et al. constructed a supramolecular hydrogel loading oxidized dextran, anti-angiogenic nanomedicines and TAMs-reprogramming polyTLR7/8a nano-regulators for orthotopic liver cancer therapy [71]. Injectable hydrogels for local drug delivery have been recently developed for tumor recurrence after surgery [72,73]. A postoperative tumor recurrence model was established, as shown in Figure 9H. When the volume of tumors reached approximately 200 mm^3^, primary tumors were partially removed and were evaluated using in vivo imaging systems for the next 14 days. As shown in Figure 7I, tumor recurrence took place on day 7 (Figure 9I). On day 14, the ZNIDL gel + Laser treatment group always had a very low bioluminescence while recurrent tumors increased over time in the other groups (Figure S29). As expected, the ZNIDL gel with laser irradiation reduced the local tumor recurrence rate. Thus, the controlled drugs release within the gel at the specific site was an effective method for preventing postoperative tumor recurrence.

## 4. Conclusions

Multifunctional nanoparticles are of significant importance for synergistic multimodal antitumor activity. In the present research, ZNIDL NPs were prepared and characterized. The as-prepared ZNIDL NPs were decomposed into cytotoxic Zn^2+^, leading to a tumor-specific release of ICG and DOX. The ZnO of the ZNIDL NPs provided oxygen and reactive oxygen species for photodynamic therapy. The ZNIDL NPs had a good photodynamic and photothermal performance. The ZNIDL NPs had a time-dependent cellular uptake and antitumor efficacy in the 4T1 cells. The ZNIDL NPs exhibited higher CRT, ATP and HMGB1 levels to induce immunogenic cell death effect. The ZNIDL NPs induced favorable antitumor immune responses, including DC maturation and T cell activation. The in vivo antitumor activity of ZNIDL NPs not only suppressed tumor proliferation but also reduced tumor metastasis and recurrence. Therefore, this study could provide an efficient approach for designing multifunctional nanoparticles for synergistic multimodal antitumor therapy.

## Data Availability

The data that support this study are available from the corresponding authors upon reasonable request.

## Supplementary Materials

The following supporting information can be downloaded at: https://www.mdpi.com/article/10.3390/jfb15020035/s1. Supplementary methods: Table S1. Concentrations of the endocytosis inhibitors. Figure S1. Characterization of ZnO NPs. Figure S2. Characterization of ZnO-NH_2_ NPs. Figure S3. TEM image of ZNIDL NPs. Figure S4. Fluorescence spectra of ZNIDL NPs. Figure S5. Fluorescence spectra of ICG adding ZnO NPs. Figure S6. DOX release from ZNIDL NPs in vitro. Figure S7. Quantitative O_2_ generation of ZnO NPs with H_2_O_2_ solutions. Figure S8. Detection of O_2_ generation from ZnO NPs with H_2_O_2_ solutions. Figure S9. Photothermal stability of ZNIDL NPs. Figure S10. Photothermal photographs of ZNIDL NPs. Figure S11. Intracellular ROS generation in 4T1 cells. Figure S12. Characterization of the gel. Figure S13. Temperature changes in the tumor in mice via peri-tumoral injection with ZNIDL gel and exposed to laser irradiation. Figure S14. In vivo photothermal photographs under laser irradiation. Figure S15. Individual tumor growth curves in 4T1 tumor-bearing mice. Figure S16. Photographs of lung tissues in 4T1 tumor-bearing mice. Figure S17. TUNEL staining in 4T1 tumor-bearing mice. Figure S18. HIF-1α immunofluorescence staining in 4T1 tumor-bearing mice. Figure S19. H&E staining in 4T1 tumor-bearing mice. Figure S20. Body weight in 4T1 tumor-bearing mice. Figure S21. Gating strategy to sort in vivo matured DCs. Figure S22. Gating strategy to sort in vivo T lymphocyte cells. Figure S23. Percentages of CD3^+^ CD8^+^ T cells within spleens. Figure S24. Immunofluorescence staining of CD3^+^ CD8^+^ T cells. Figure S25. Immunofluorescence staining of CRT. Figure S26. Immunofluorescence staining of HMGB1. Figure S27. Individual tumor growth curves in the 4T1 bilateral tumor model. Figure S28. Body weights in the 4T1 bilateral tumor model. Figure S29. Radiance intensities in the postoperative tumor recurrence model.
