# Supplementary material for: Multifunctional ZnO@DOX/ICG-LMHP Nanoparticles for Synergistic Multimodal Antitumor Activity
Source: J Funct Biomater. 2024 Jan 30;15(2):35. doi: 10.3390/jfb15020035 (PMC10889406; doi:10.3390/jfb15020035)
Supplement: Supplementary file 1 [file jfb-15-00035-s001.zip › jfb-2800634-supplementary.pdf]

Supplementary Methods

Preparation of ZNIDL NPs

ZnO NPs were synthesized using a sol–gel method. Firstly, $Zn(OAc)_2{\cdot}2H_2O$ (1.09 g) and NaOH (0.368 g) were dissolved in ethanol. The solutions were mixed by rapidly stirring and reacted until the solution was clear. Zinc oxide was obtained by precipitation of heptane. The sol–gel method was cheap and safe to obtain uniform ZnO NPs.

Photodynamic and Photothermal Effect of ZNIDL NPs

Dichloro-dihydro-fluorescein diacetate (DCFH-DA) was used to detect the total ROS generation. ZNIDL NPs were mixed with a DCFH solution (10 μM) and irradiated with 808 nm (1 W/cm^2, 5 min). The fluorescence intensity was detected using fluorescence spectrophotometry (λex = 504 nm, λem = 529 nm).

The compound 1,3-diphenylisobenzofuran (DPBF) was used to detect singlet oxygen ($^1O_2$). DPBF was oxidized by $^1O_2$ and caused the absorption to decrease at 410 nm. DPBF (1 mg/mL in DMSO) was added to a PBS solution containing ICG and ZnO NPs with or without H_2O_2 under laser irradiation for various times. The absorption at 410 nm was measured.

To evaluate the photothermal effect of the ZNIDL NPs, a 0.5 mL dispersion at different ICG concentrations (0, 2.5, 5, 10, 20 μg/mL) was irradiated under laser irradiation (808 nm, 1.0 W/cm^2) for 5 min. In a second set of experiments, a given ICG concentration (10 μg/mL) was irradiated under different power intensities (0.2, 0.5, 1, 1.5, 2 W/cm^2) for the laser irradiation. The photothermal effect of the ZNIDL NPs (10 μg/mL), ICG (10 μg/mL) and four control samples (H_2O, DOX, ZnO and LMHP) were also investigated. The photothermal conversion of the ZNIDL NPs was determined using the same method. The temperature changes in the samples were monitored using a thermal infrared imaging camera (FOTRIC 230, Fotoric Thermal Intelligence, China).

Intracellular Behavior of ZNIDL NPs

The 4T1 cells were incubated in confocal dishes or 12-well plates for 24 h. Then, ZNIDL NPs with 8 μM DOX were added. The endocytosis pathway was determined by the pre-administration of inhibitors, as shown in Table S1. The fluorescence intensity of DOX was detected using a confocal laser scanning microscope (ZEISS, Germany) and flow cytometer (Beckman Coulter, USA) (λex = 488 nm and λem = 560 nm for DOX).

Table S1. Concentrations of the endocytosis inhibitors.

Inhibitors	Function	Concentration
Filipin	Inhibitor of lipid raft	0.5 μg/mL
Hypertonic sucrose	Inhibitor of clathrin	0.4 M
Chlorpromazine (CPZ)	Inhibitor of clathrin	30 μM
EIPA	Inhibitor of macropinocytosis	20 μM

In Vitro Antitumor Activity of ZNIDL NPs

The Sulforhodamine B (SRB) assay was used to evaluate the antitumor activity of the ZNIDL NPs in vitro. The 4T1 cells were incubated in 96-well plates for 24 h. Then, ZNIDL NPs with 0, 0.1, 1 and 4 μM DOX were added. The absorbance was detected at 540 nm using the ELx808 plate reader (BioTek, USA). The cell viability was calculated using the following formula. Survival % = (A_{540nm} for the treated cells/A540 nm for the control cells) × 100%, where A_{540nm} is the absorbance value.

The Annexin V-FITC/DAPI Apoptosis Kit was used to evaluate cell apoptosis using a flow cytometer. In brief, the 4T1 cells were incubated in 12-well plates for 24 h. Then,

ZNIDL NPs with 1 μM DOX were added. The cell apoptosis rates were equal to the sum of the early apoptosis rate and late apoptosis rate.

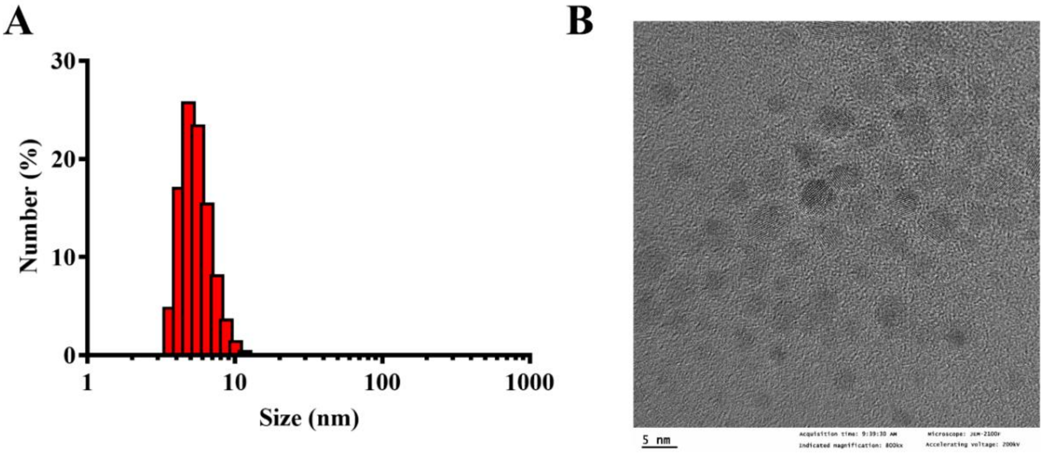

Figure S1. Characterization of ZnO NPs. (**A**). ZnO NPs particle size. (**B**). ZnO NPs TEM image.

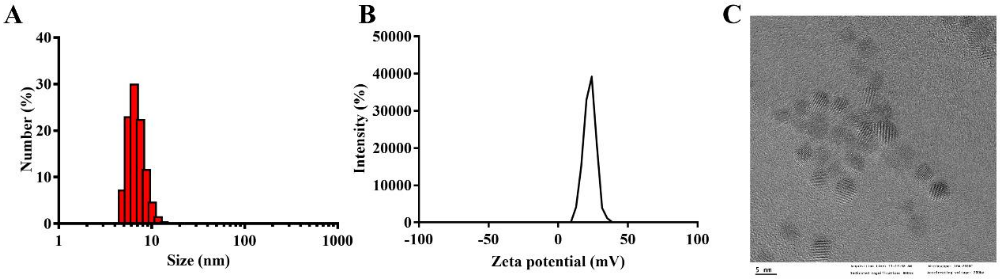

Figure S2. Characterization of ZnO-NH_2 NPs. (**A**). ZnO-NH_2 NPs particle size. (**B**). ZnO-NH_2 NPs zeta potentials. (**C**). ZnO-NH_2 NPs TEM image.

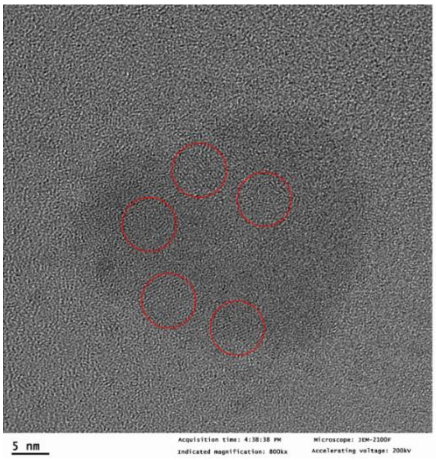

Figure S3. ZNIDL NPs TEM image.

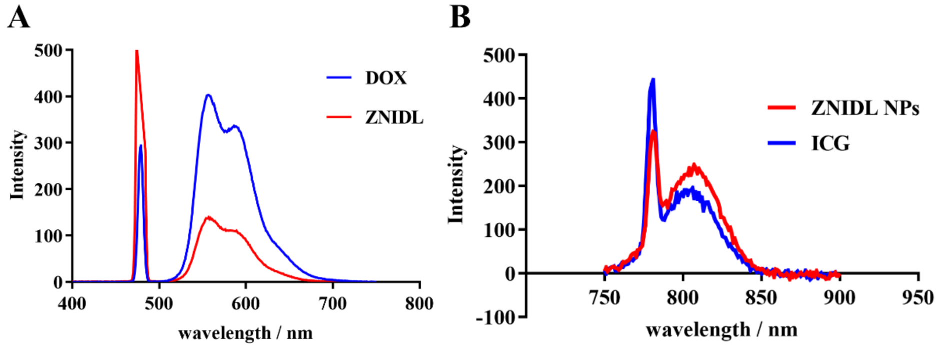

Figure S4. Fluorescence spectra of ZNIDL NPs. (**A**). The excitation wavelength at 480nm. (**B**). The excitation wavelength at 780nm.

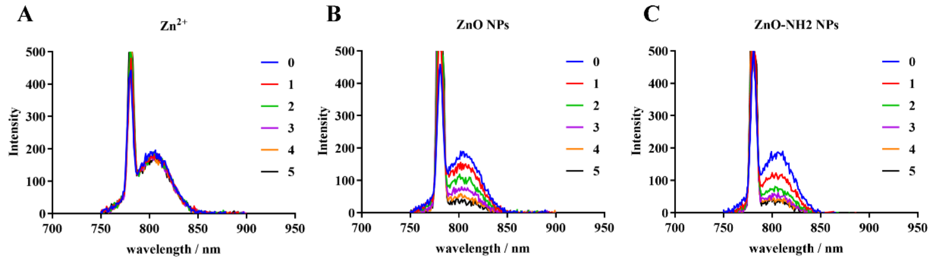

Figure S5. Fluorescence spectra of ICG adding ZnO NPs. (**A**). Zn^{2+} ions. (**B**). ZnO NPs. (**C**). ZnO-NH_2 NPs.

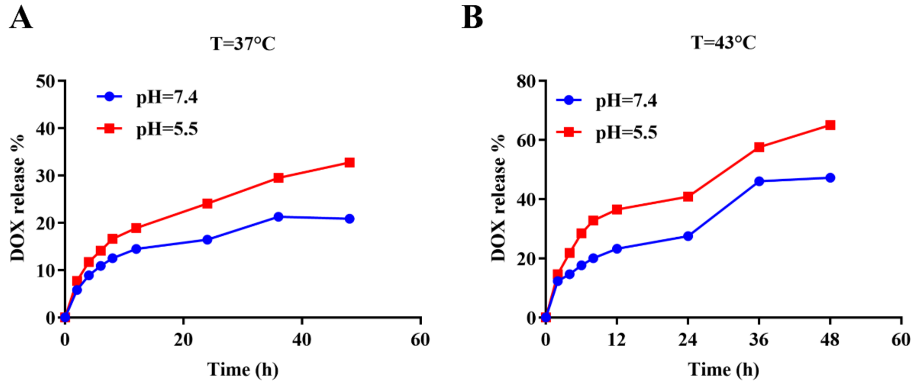

Figure S6. DOX release from ZNIDL NPs in vitro. (**A**) The temperature of 37 °C. (**B**). The temperature of 43 °C.

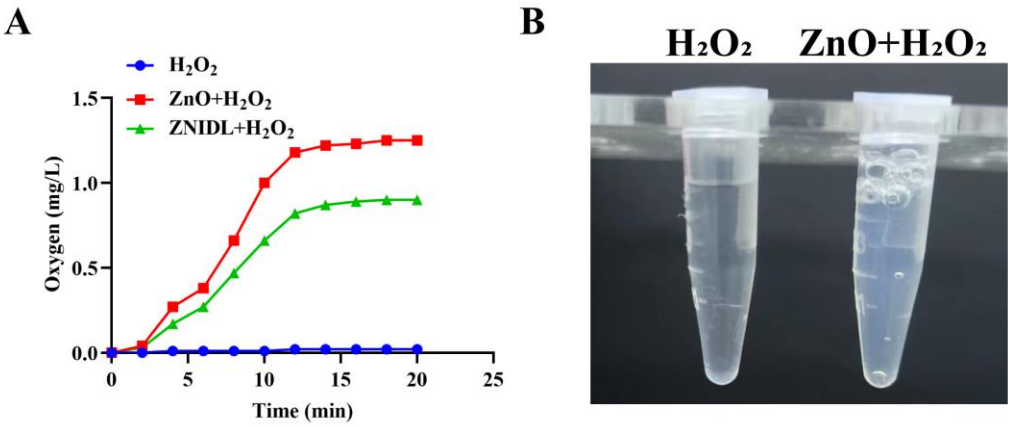

Figure S7. Quantitative O_2 generation of ZnO NPs with H_2O_2 solutions. (**A**). Quantitative O2 generation. (**B**). Photographs.

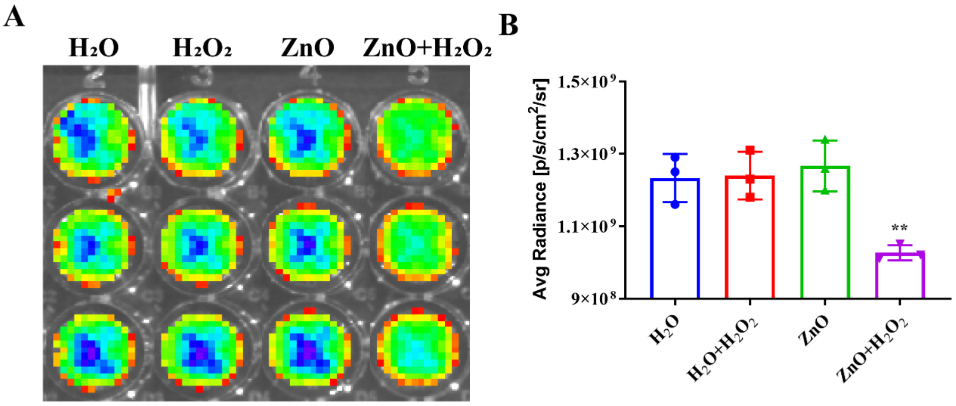

Figure S8. Detection of O_2 generation of ZnO NPs with H_2O_2 solutions. (**A**). Photographs. (**B**). Average radiance intensities.

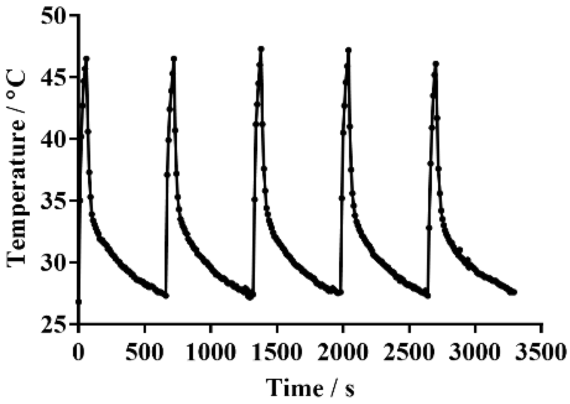

Figure S9. Photothermal stability of ZNIDL NPs.

A

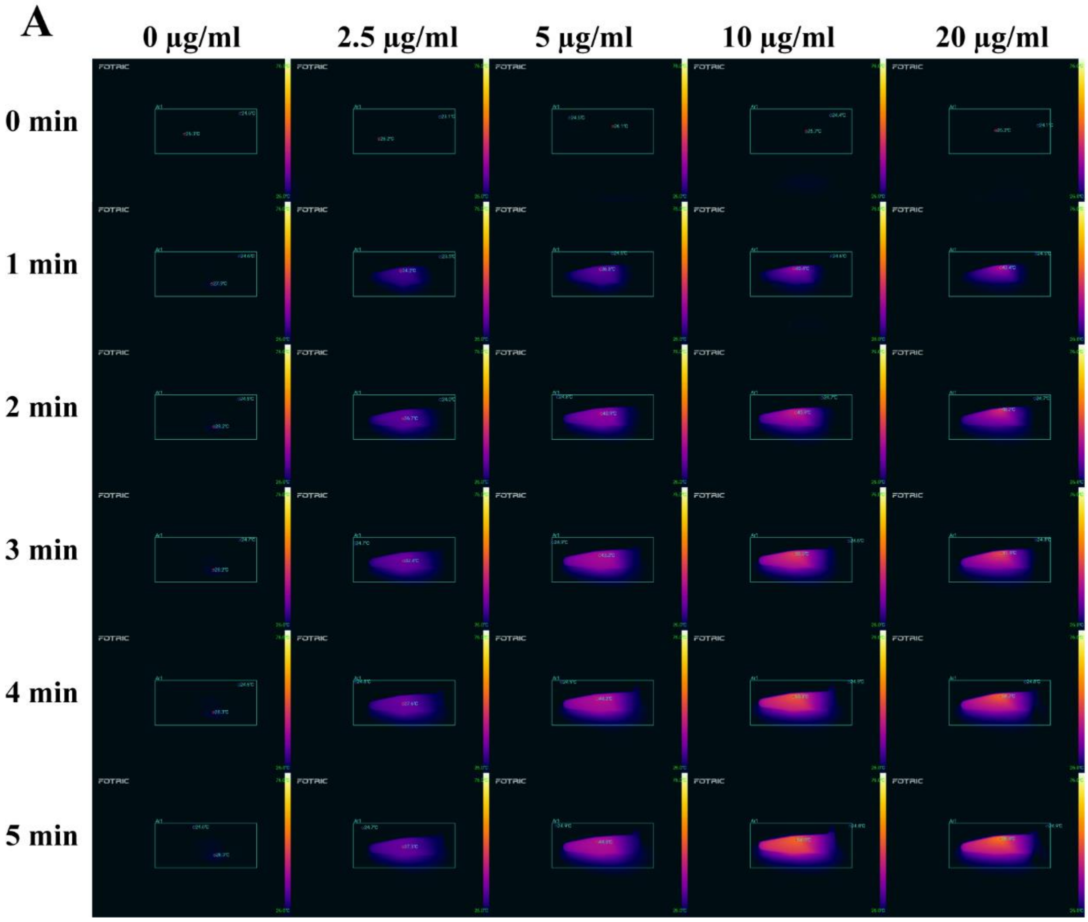
0 μg/ml
2.5 μg/ml
5 μg/ml
10 μg/ml
20 μg/ml
0 min
1 min
2 min
3 min
4 min
5 min

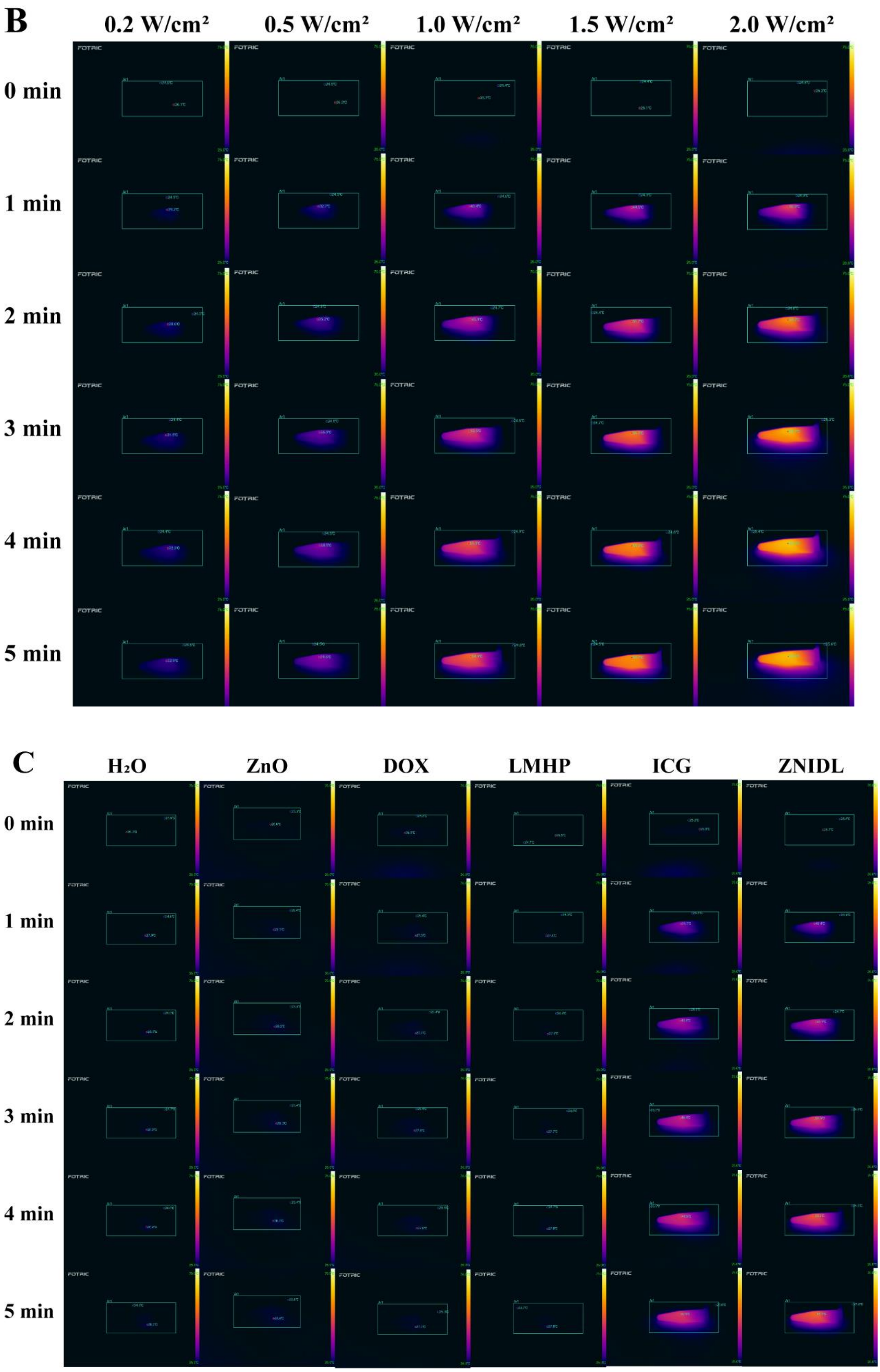
B
0.2 W/cm²
0.5 W/cm²
1.0 W/cm²
1.5 W/cm²
2.0 W/cm²
0 min
1 min
2 min
3 min
4 min
5 min
C
H2O
ZnO
DOX
LMHP
ICG
ZNIDL
0 min
1 min
2 min
3 min
4 min
5 min

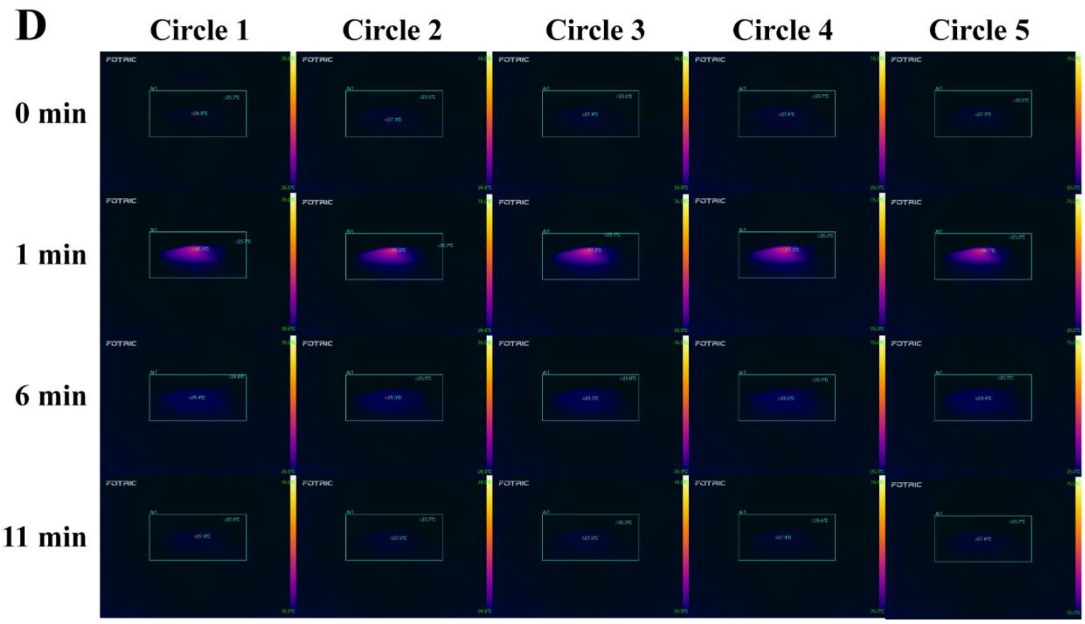

Figure S10. Photothermal photographs of ZNIDL NPs. (**A**). Temperature photographs for ZNIDL NPs at different concentrations. (**B**). Temperature photographs for ZNIDL NPs with different power intensities. (**C**). Temperature photographs for ZNIDL NPs with H2O, DOX, ZnO NPs, LMHP, and ICG used as control samples. (**D**). Temperature photographs of ZNIDL NPs over five on/off cycles of laser irradiation.

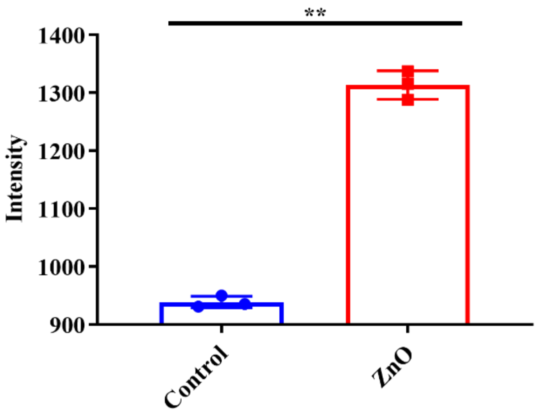

Figure S11. Intracellular ROS generation. ** $p < 0.01$ vs control treatment group.

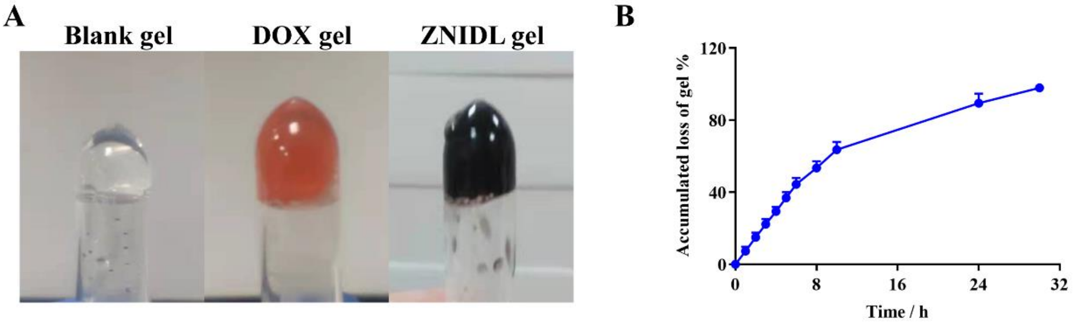

Figure S12. Characterization of gel. (**A**). The images of gel. (**B**). Accumulated loss of gel.

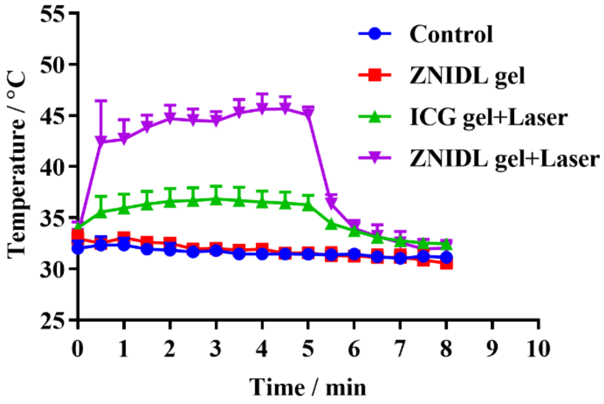

Figure S13. Temperature changes of the tumor in mice via peri-tumoral injection with ZNIDL gel and exposed to laser irradiation.

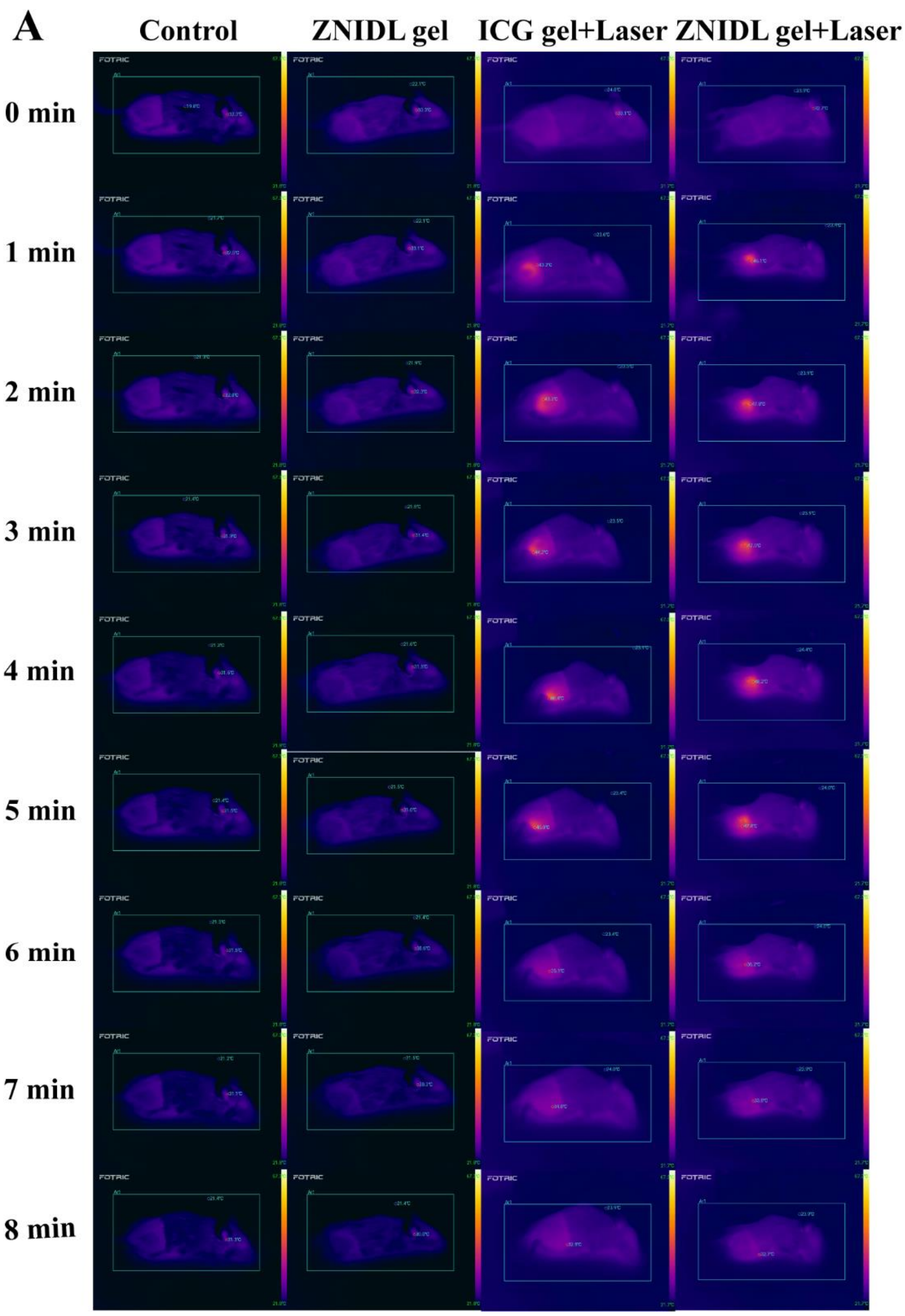
A
Control
ZNIDL gel
ICG gel+Laser
ZNIDL gel+Laser
0 min
1 min
2 min
3 min
4 min
5 min
6 min
7 min
8 min

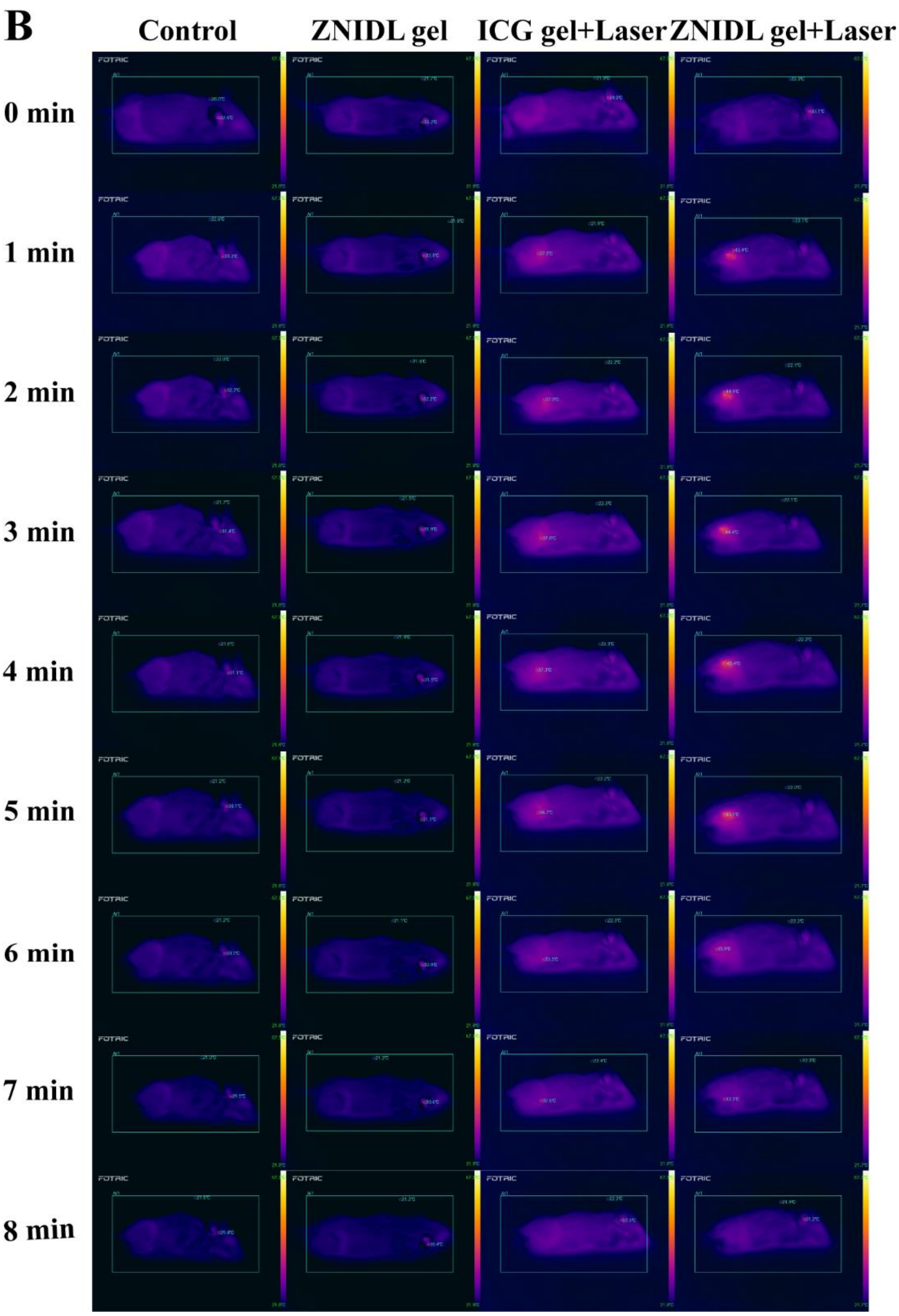

Figure S14. In vivo photothermal images under laser irradiation. (**A**). The first time. (**B**). The second time.

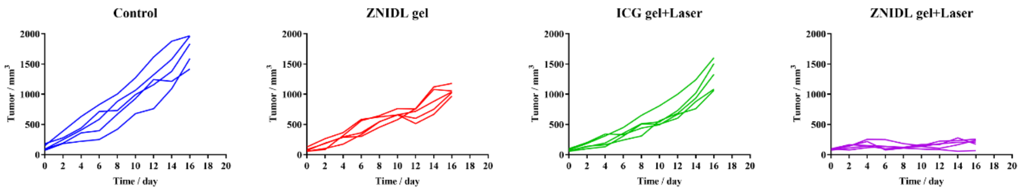

Figure S15. Individual tumor growth curves in 4T1 tumor-bearing mice.

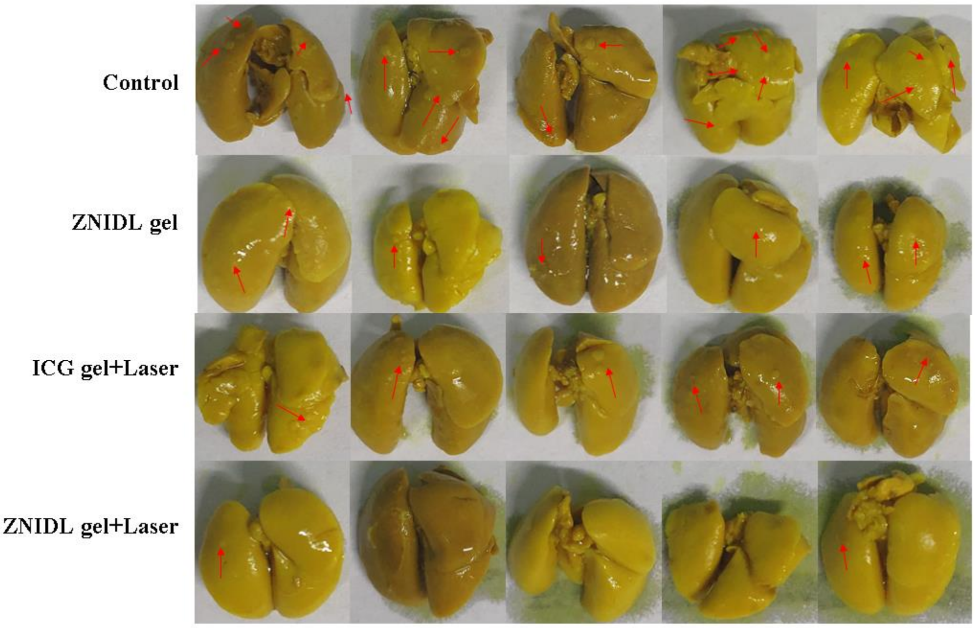

Figure S16. Photographs of lung tissues in 4T1 tumor-bearing mice.

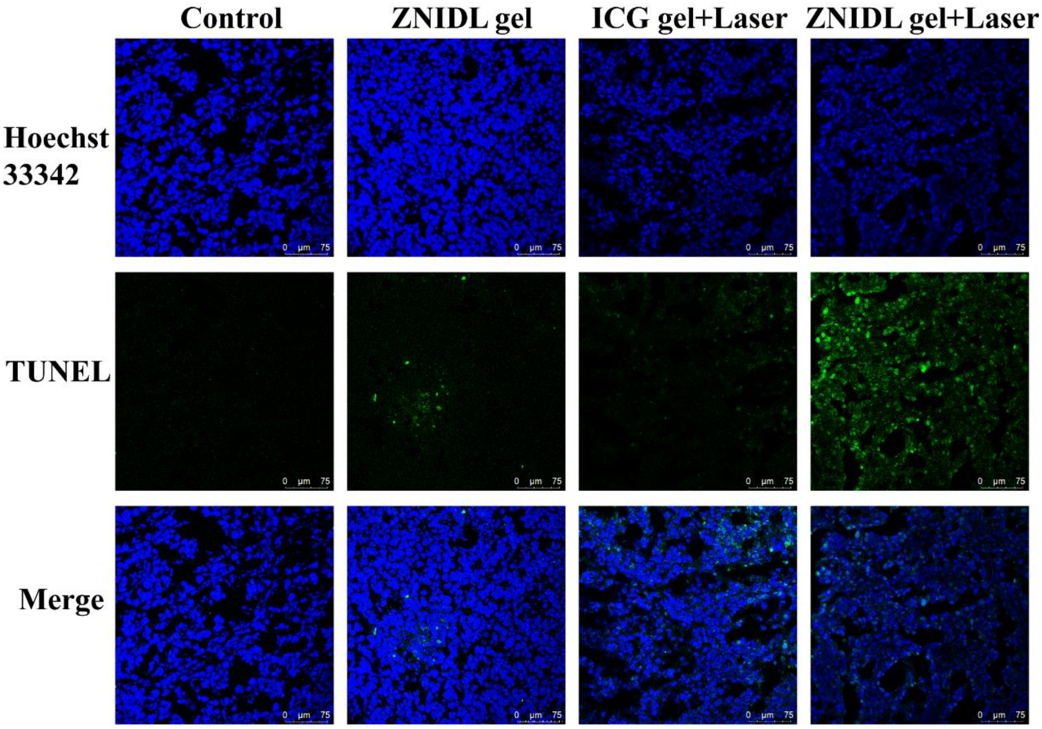

Figure S17. TUNEL staining in 4T1 tumor-bearing mice. (Scale bars = 75 μm).

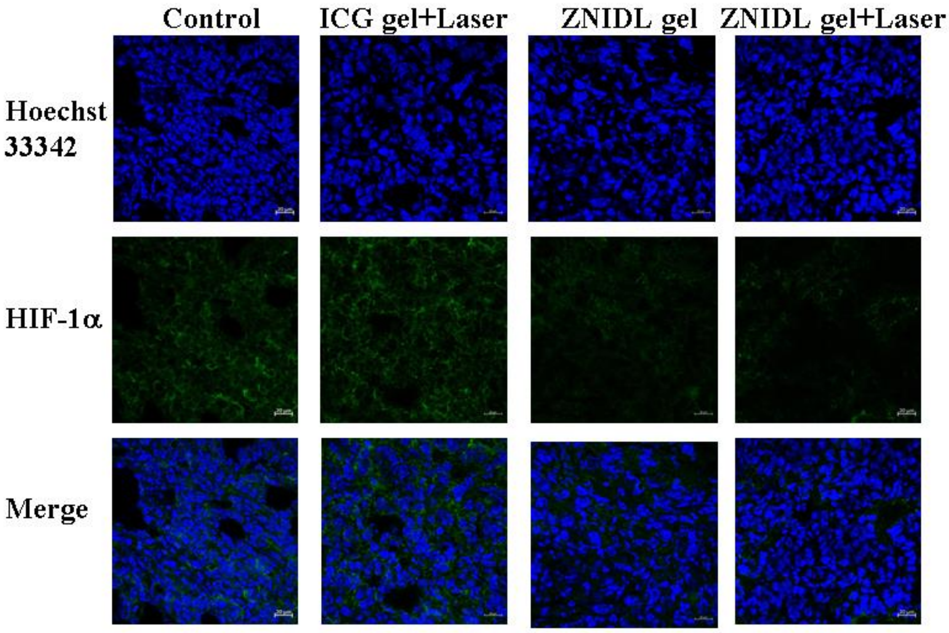

Figure S18. HIF-1α immunofluorescence staining in 4T1 tumor-bearing mice. Scale bars = 20 μm.

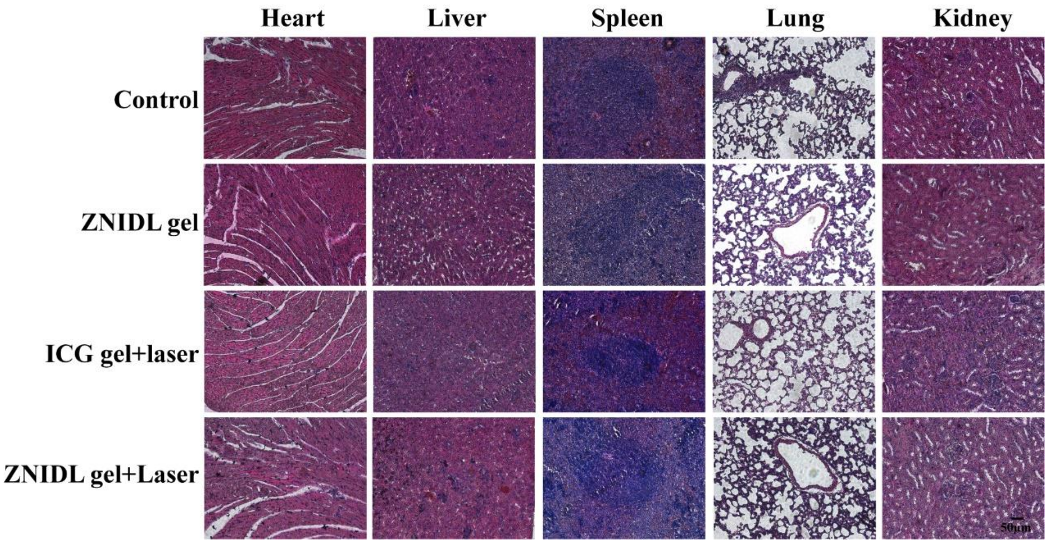

Figure S19. H&E staining in 4T1 tumor-bearing mice. Figure S20. Body weight in 4T1 tumor-bearing mice. Scale bar = 50 μm.

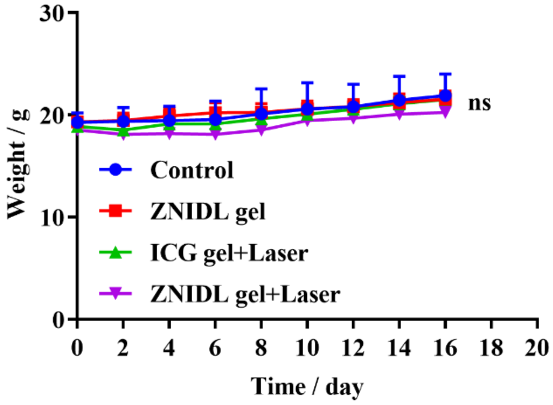

Figure S20. Body weight in 4T1 tumor-bearing mice (n = 5).

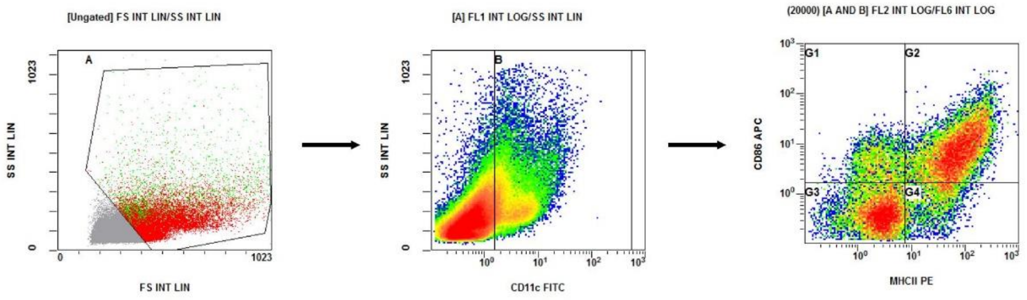

Figure S21. Gating strategy to sort in vivo matured DC cells. The lymph node from mice treated with control group was used as the example.

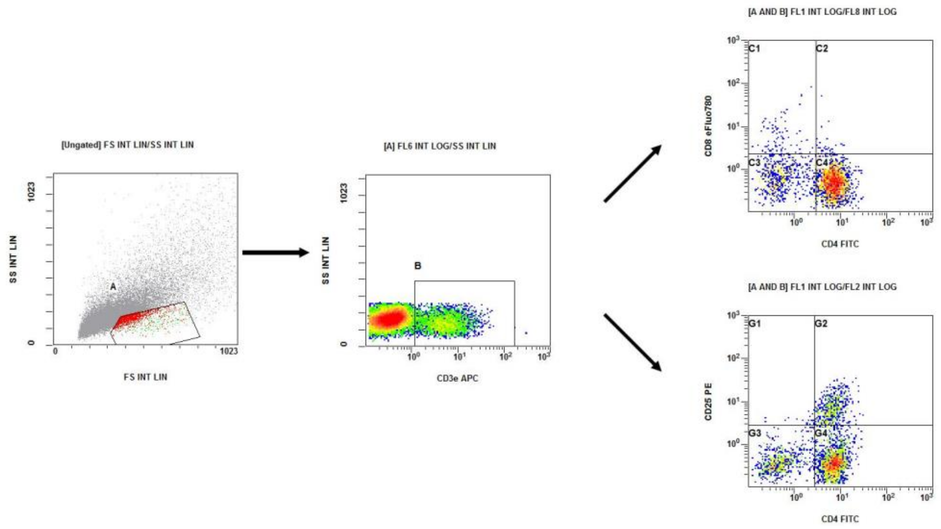

Figure S22. Gating strategy to sort in vivo T lymphocyte cells. The tumor from mice treated with control group was used as the example.

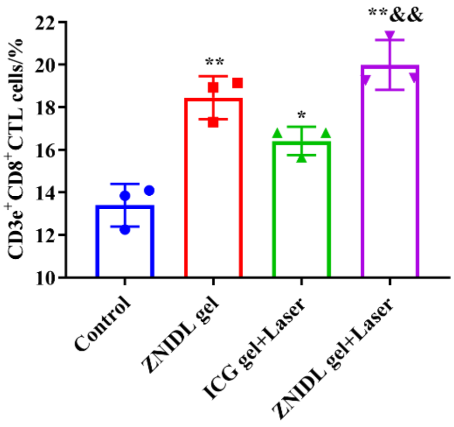

Figure S23. Percentages of $CD3^+$ $CD8^+$ T cells within spleens. * $p < 0.05$ vs control treatment group. ** $p < 0.01$ vs control treatment group. *&&* $p < 0.01$ vs ICG+Laser treatment group.

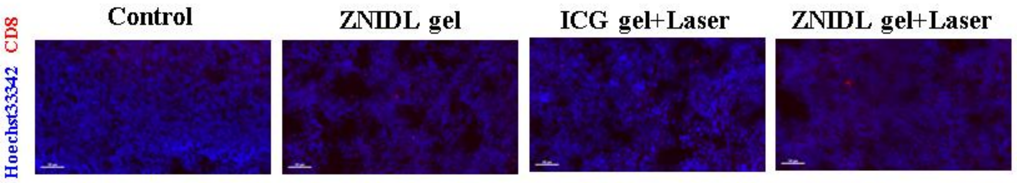

Figure S24. Immunofluorescence staining of CD3+ CD8+ T cells. Scale bar = 50 μm.

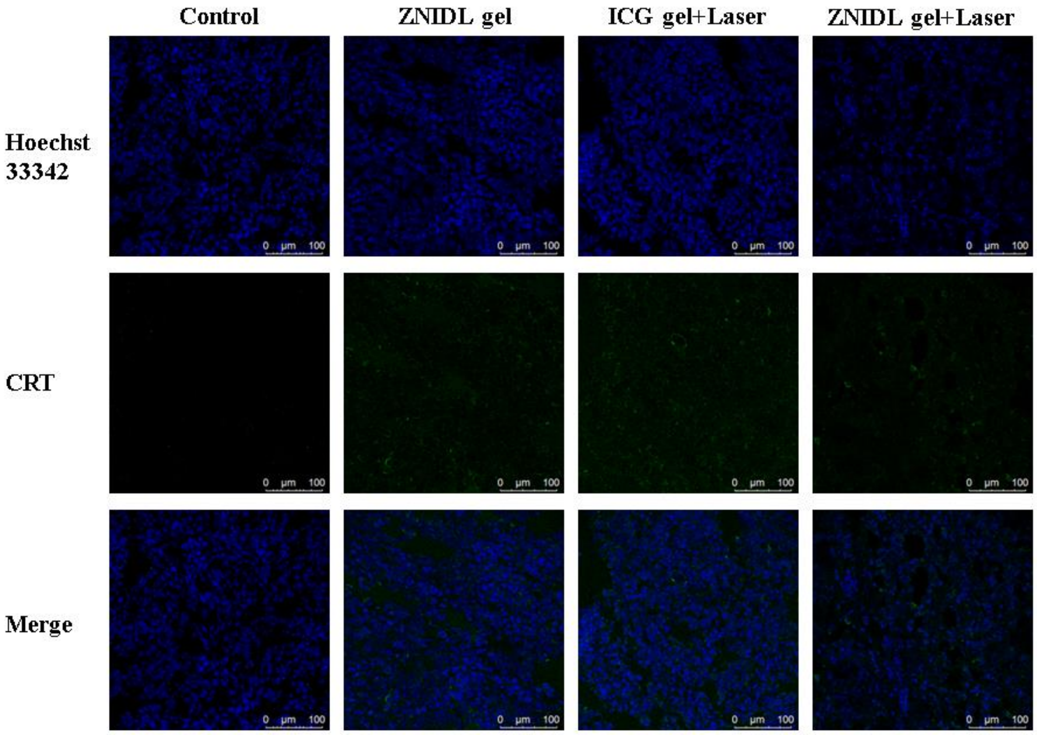

Figure S25. Immunofluorescence staining of CRT. Scale bar = 100 μm.

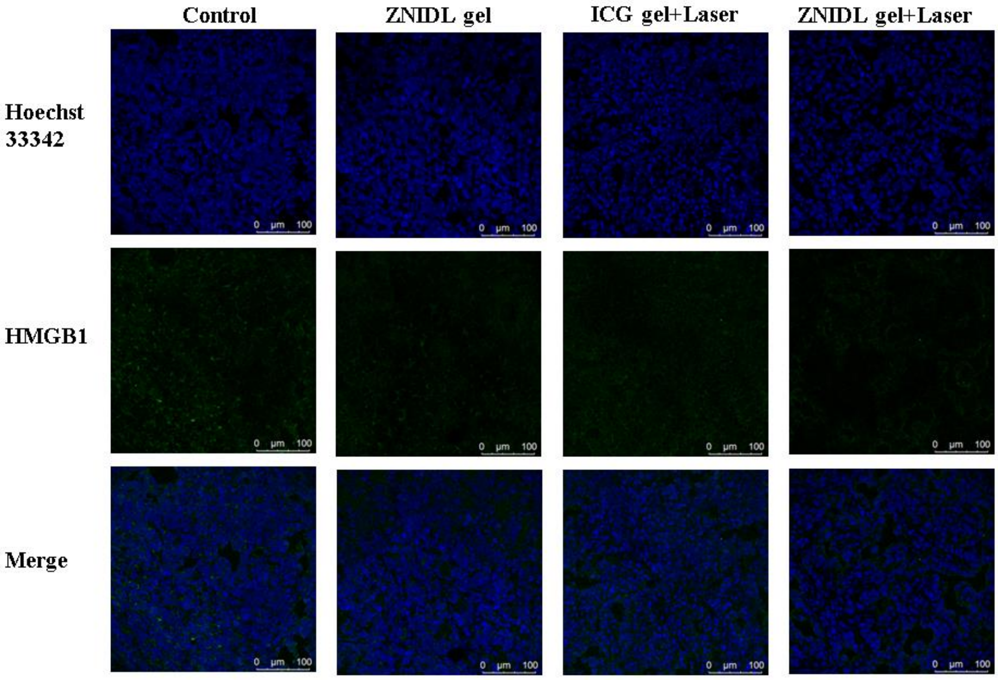

Figure S26. Immunofluorescence staining of HMGB1. Scale bar = 100 μm.

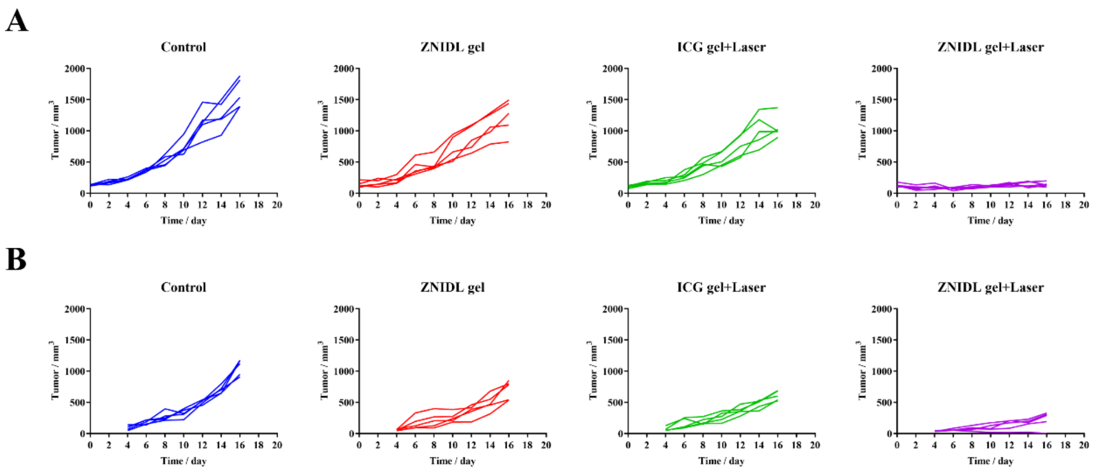

Figure S27. Individual tumor growth curves in 4T1 bilateral tumor model. (**A**). The primary tumors. (**B**). The distant tumors.

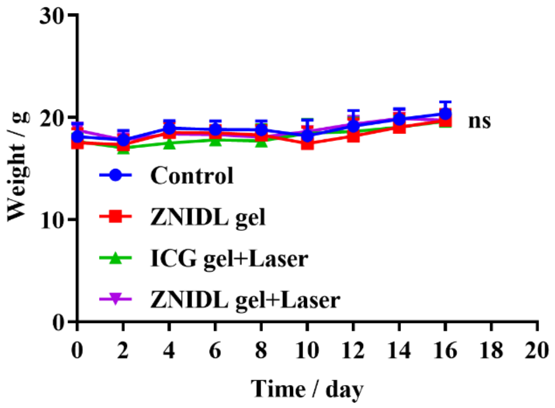

Figure S28. Body weight in 4T1 bilateral tumor model (n = 5).

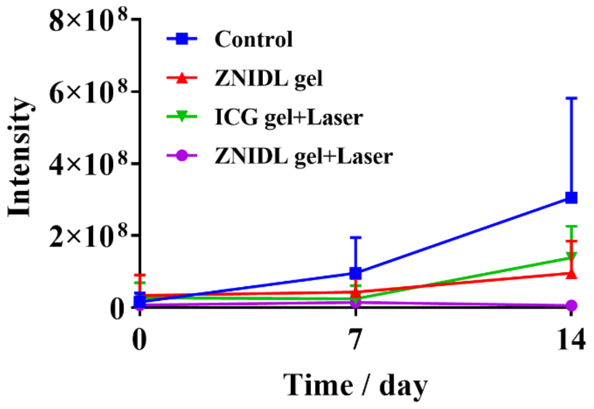

Figure S29. Radiance intensities in postoperative tumor recurrence model (n = 3).
